# Adoptive T Cell therapy for cancer and autoimmune disease: current advances, challenges, and future directions

**DOI:** 10.3389/or.2026.1905358

**Published:** 2026-09-16

**Authors:** Qiu-Hong Bao, Angel Wu, Jianming Pang, Junwei Wang, Qingqing Chen, SuWen Wu

**Affiliations:** 1 Department of Medical Oncology, Tiantai County People’s Hospital, Taizhou, Zhejiang, China; 2 Department of Clinical Medicine, Internal Medicine, Zhejiang Chinese Medical University, Hangzhou, Zhejiang, China

**Keywords:** autoimmune diseases, cancer, cancer treatment, chimeric antigen receptor T cell (CAR-T) therapy,, T cell receptor-engineered T cell (TCR-T) therapy

## Abstract

Adoptive T Cell Therapy (ACT) is a kind of immunotherapy that eliminates target cells by autologous T cells. Since the mechanism of adoptive immune was elucidated, ACT has been developed for more than half a century. As one of ACTs, chimeric antigen receptor (CAR)-T cell therapy produced remarkable clinical responses in some hematological malignancies that is considered revolutionary strategy of immunotherapy. However, the effects of CAR-T cell therapy in solid tumors are hindered by immunosuppressive environment and limit of CAR to only recognize antigens on the cell surface. T cell receptor-engineered T cell (TCR-T) therapy is another type of ACT that the TCR is programmed to match crucial tumor antigens according to next-generation sequencing and bioinformatics analysis. The therapy overcomes the obstacles in solid tumor treatment by CAR-T, while there are also some problems dampening the response such as tumor immune escape, T cell exhaustion and toxicity. Beyond oncology, ACT is also applied in autoimmune diseases for it can specifically target and kill related immunocytes. In this review, we summarize the characteristics and clinical trials of CAR-T and TCR-T for cancer and autoimmune diseases. Differences, challenges and prospect of these ACTs are also described for reference to novel strategy of ACT.

## 1 Introduction

T lymphocytes (T cells) are the mainstays of adaptive immunity, undertaking prevention of pathogen invasion and tumor growth to maintain human health. Natural senescence and experimental exposomes result in disorder of immune homeostasis by gene damage and epigenetic alternations, leading infectious diseases, cancers and autoimmune diseases. To recover immune balance, syngeneic T cells have been used to transfer into patients for treatment of immune related diseases especially cancers, which is termed as “adoptive T cell therapy (ACT)”.

As a kind of immunotherapy, ACT has been studied and applied for over half a century. When the functions of T cells were gradually unveiled from 1960s, their ability of mediating allo-graft rejection attracted attention for exploring the mechanism of cell killing and clinical application of tumor elimination. The animal experiments demonstrated transfer of syngeneic lymphocyte from tumor-immunized individual modestly inhibited the growth of tumor (1, 2), establishing the foundation of ACT. The application of ACT was facilitated by the elucidation of interleukin-2 (IL-2) function that provided an approach to maintain and proliferate T cells *ex vivo* (3). Tumor infiltrating lymphocytes (TILs) and lymphokine activated killer cells (LAKs) were the main components of the first generation of ACT (4, 5). However, their effects were limited, since the period of TIL amplification was too long for patients with rapid progressive tumors, and the lymphocytes isolated from tumor tissues were not all tumor targeting (6). The advance of genetic engineering and cell culture technology made possible for modified T cells with shorter proliferation time and better tumor-specificity. In 1989, G. Gross et al first designed a kind of genetically modified T cells with synthetic receptors to detect and eliminate tumor cells, which was called chimeric antigen receptor T cell (CAR-T) therapy (7). CAR-T has been proven safe and effective against hematological cancer, yet, there are many obstacles in solid tumor treatment by CAR-T owing to the complicated microenvironment (8). T cell receptor-engineered T cell (TCR-T) therapy is another type of ACT that the TCR is programmed to match crucial tumor antigens according to next-generation sequencing and bioinformatics analysis (9). It is free from the limit of surface tumor antigen recognition because its TCR can recognize intracellular antigens presented by human leukocyte antigens (HLA). Many clinic trials are being conducted to assess the safety and efficiency of TCR-T for hematological and solid tumor treatment (10).

CAR-T and TCR-T are the main streams of ACT nowadays. Several CAR-T cell products approved by Food and Drug Administration (FDA) have appeared on market, including Kymariah (Tisagenleleucel) (11), Yescarta (Axicabtagene Cilloleucel) (12), Tecartus (Brexucabtagene Autoleucel) (13), Breyanzi (Lisocabtagene Maraleucel) (14), Abecma (Idecabtagene Vicleucel) (15), and Carvykti (Citacabtagene Autoleucel) (16). TCR-T has also shown effectiveness against tumors in preclinical and clinical trials (9, 17). Besides, ACT has been used for autoimmune diseases by eliminating auto-reactive immunocytes and regulating immune microenvironment (18). In this review, we will outline the frontier progress, clinical application challenges and future directions of ACT in tumor and autoimmune disease.

## 2 Characteristics of car-t and TCR-T cells

### 2.1 The structures of CAR

CAR-T cells are genetically modified T cells with CAR expression on their surface. CAR is single chain protein and generally divided by three segments: (1) the extracellular structural domain including a monoclonal antibody against target molecules; (2) the transmembrane domain; (3) the intracellular structural domain responsible for transducing signals (19).

The extracellular structural domain is usually single-chain variable fragment (scFv) made by heavy chain (VH) and light chain (VL) binding by flexible junctions (20). Although the immunogenicity of scFv is highly diminished in comparison to full-length antibody, synthetic linker between VH and VL in scFv can still induce neutralizing antibodies of host that abates the effectiveness of CAR (21, 22). Furthermore, CAR-T exhaustion may occur resulting from self-aggregation of VH and VL in scFv (namely, CAR aggregation) leading to affinity loss (23). As alternative of scFv, a single variable heavy chain (VHH) structure derived from sharks and the Camelidae family have been used as a strong competitor of scFv for CAR (24). VHH, also called nanobodies, only comprises of variable heavy chain that avoid immune response against synthetic linker and CAR aggregation with similar specificity and affinity to target antigens compared with scFv (25). The smaller size of nanobodies allow them to be designed as tandem CARs (TanCARs) that can target different antigens in single chain with relative high efficiency of viral transfection and CAR expression (26). Long complementarity-determining region (CDR) 3 is another advantage of nanobodies that enabling them to target particular epitopes which full-length antibodies and scFv can’t (27). Nanobody-based CAR-T cells have been shown effective in treatment of cancers targeting vascular endothelial growth factor receptor 2 (VEGFR2) (28), human epidermal growth factor receptor 2 (HER2) (29), prostate-specific membrane antigen (PSMA) (30, 31), CD33 (32), Epidermal growth factor receptor (EGFR) (33, 34), CD20 (35) and Mucin 1 (MUC1) (36), indicating their great potential against cancer.

Mainly responsible for anchoring CAR on T cell membrane, the transmembrane domain usually originates from CD3ζ, CD4, CD8α, or CD28, and influence signal transduction (37). CD3ζ transmembrane domain is apt to induce CAR-mediated T cell activation while its stability is relative weak (38). In comparison, CD28 transmembrane domain can ignite more violet immune response with high level of inflammatory cytokines, however, it elevates susceptibility to activation induced cell death (AICD) (39). For effective signal transduction, the transmembrane domain needs to be assembled with corresponding intracellular domain.

The vast majority of intracellular structural domains of CARs comprise of CD3ζ which conducts signal transduction by immune receptor tyrosine based activation motifs (ITAMs) activation and following phosphorylation of ZAP70 that trigger immune response (40). The first-generation CARs were assembled CD3ζ structure in intracellular domain without co-stimulatory regions limiting the intensity immune response evoked (41). The tandem of CD28 or 4-1BB and CD3ζ domains were used in the second-generation CARs that achieved the enhancement of proliferation ability and cytotoxicity (42). The third generation simultaneously concatenates the two co-stimulatory domains CD28 and 4-1BB with CD3ζ, while the effect elevation needs further study (43–45). To further promote CARs’ efficiency, cytokine secretors or immune checkpoint inhibitors were inserted between co-stimulatory regions and CD3ζ domains forming fourth-generation CARs (namely, T-cells redirected for universal cytokine killing, TRUCK) that exhibit heightened potency and resistant to immunosuppressive factors compared to former CARs (46). Bulit on the second-generation CARs, fifth-generation CARs were made by adding the intracellular part and transcription (STAT) 3/5 binding pattern of IL-2 receptor β chain between co-stimulatory regions and CD3ζ domains. This insertion is a mimic of IL-2/IL-2R signal realizing the activation of the 1st signal (MHC-TCR signal), 2nd signal (co-stimulatory signal) and 3rd signal (cytokine) by only the interaction between extracellular domain and target antigens (47).

### 2.2 The structures of TCR of TCR-T

As the core effector cells of adaptive immunity, αβ T lymphocytes are boosted by engaging peptide-MHC (pMHC) ligands through T cell receptor (TCR). The TCR of αβ T cells is a heterodimer composing of α and β chain with the variable (V) and constant (C) domain of immunoglobulin (Ig)-like structure, membrane-proximal connecting peptide (CP), transmembrane (TM) region, and short cytoplasmic tail. Anchored to the cell membrane by a transmembrane region, followed by a short cytoplasmic tail (48). V and C domains comprise of three complementary determining regions (CDR1, CDR2, CDR3). CDR3 has high diversity owing to DNA recombination of V, (D), and J segments and random modifications (P/N additions) of the gene end, while CDR1 and CDR2 have lower diversity that they are encoded within the TCR V segments (49). During antigen recognition, CDR1 and CDR2 are responsible for interacting with HLA, while CDR3 directly contacts the antigen specifically (50). CD3γε, CD3δε heterodimers and CD3ζ homodimers are required to TCR responsible for signal transduction. Each CD3γ/δ/ε subunit includes immunoreceptor tyrosine-based activation motif (ITAM) based on tyrosine (51, 52). When TCR recognize pMHC molecules, ITAMs are phosphorylated by the src kinase (Lck), recruiting protein tyrosine kinase ZAP70 by binding to phosphorylated tyrosine residues through SH2 domains (53). Subsequently, ZAP70 is phosphorylated and activated by Lck, leading phosphorylation of multiple tyrosine residues of the transmembrane protein LAT, providing binding sites for downstream signaling molecules (54). Cytotoxic T-lymphocyte antigen 4 (CTLA-4) can inhibit TCR signal transduction by dephosphorylating ZAP70 and LAT. To make T cells targeting particular antigens, reconstructed TCRs with antigen specific CDR domains are designed to express in T cells which is called TCR-T cells. The TCRs can spontaneously be assembled with CD3 molecules and efficiently mediate signal transduction (55). The structural differences between CAR and TCR are summarized in Table 1.

**TABLE 1:** The structural differences between CAR and TCR.

Factors	TCRs	CARs
Sructure	Complex transmembrane receptors, ten ITAMs, needs Co-stimulation transmission	Artificial single strand, three ITAMs. Contains Co-stimulation transmission
MHC restriction	Yes	No
Typical affinity	K_d_ 1–100 μM	K_d_ 1 nM - 1 μM
IS formation	Organized	Disorganized
FDA approval	Yes	No
Antigen types	Intracellular and extracellular	Only surface antigen

### 2.3 The differences between CAR-T and TCR-T

As engineered cell therapies, CAR-T and TCR-T cell have been dominant in ACT. They are designed for specific recognition of tumor antigens and ignite T cell-mediated anti-tumor immunity by insertion of exogenous genes. Through gene transduction and activate T cell-mediated anti-tumor protection. Differences between CAR-T and TCR-T in antigen recognition, structural composition, and other aspects determine their different therapeutic effects and application choices (Figure 1).

**FIGURE 1:**
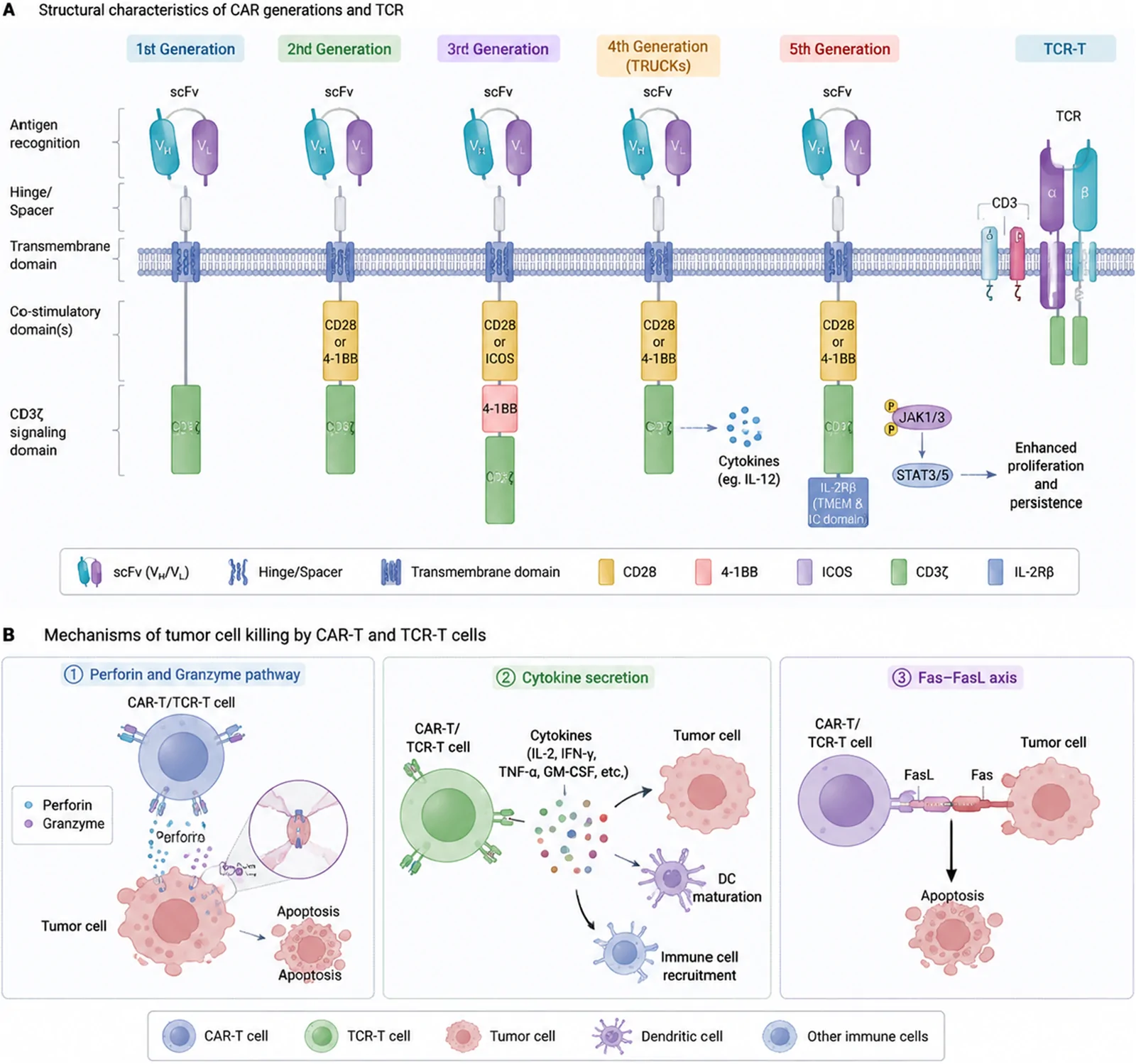
Structural characteristics and antitumor mechanisms of CAR-T and TCR-T cells. **(A)** Structural characteristics of different generations of chimeric antigen receptors (CARs) and the T-cell receptor (TCR) complex. First-generation CARs consist of an extracellular single-chain variable fragment (scFv), a hinge/spacer region, a transmembrane domain, and an intracellular CD3ζ signaling domain. Second-generation CARs incorporate one co-stimulatory domain (CD28 or 4-1BB), whereas third-generation CARs contain two co-stimulatory domains (e.g., CD28/4-1BB or ICOS/4-1BB). Fourth-generation CARs (TRUCKs or armored CARs) are engineered to secrete immunomodulatory cytokines (e.g., IL-12) upon activation. Fifth-generation CARs additionally incorporate the intracellular domain of the IL-2 receptor β-chain (IL-2Rβ), which activates the JAK1/3–STAT3/5 signaling pathway to enhance T-cell proliferation and persistence. In contrast, TCR-T cells recognize peptide antigens through the TCR/CD3 complex. **(B)** Major mechanisms of tumor cell killing mediated by CAR-T and TCR-T cells. Activated CAR-T and TCR-T cells eliminate tumor cells through (1) perforin/granzyme-mediated cytotoxicity, (2) secretion of cytokines (including IL-2, IFN-γ, TNF-α, and GM-CSF), which promote dendritic cell maturation and immune cell recruitment, and (3) Fas–FasL-mediated apoptosis. This figure is an original schematic illustration created by the authors based on information reported in the cited literature. No previously published figure was reproduced or adapted.

CAR-T and TCR-T differ in their structural composition. CAR is an artificial receptor with antigen binding domains, co stimulatory factor and CD3 ζ tandem connection (56). The combination produces some conformations that are easily considered as foreign molecules which induce graft-versus-host disease (GVHD) (57). Although TCRs on TCR-T are not natural, only variable regions of α and β chain are artificially designed with relative lower immunogenicity (58). TCRs can naturally bind to CD3 and mediate signal transduction by more ITAMs than CAR, and they can assemble co-stimulatory molecules for T cell activation although there is no component for co-stimulatory effects in TCRs.

Differences in antigen recognition and signal transmission determine the indications of CAR-T and TCR-T cells. Antigen recognition of CAR-T relies on antigen-antibody reaction, facilitating target to tumor cells with low MHC expression (59). However, the extracellular domain of CAR can only bind to cell surface antigens resulting in many obstacles in therapy for solid tumors with little particular extracellular antigen expression (60). Endogenous or engineered TCRs recognize antigens presented by HLA molecules that includes intracellular antigens. TCR-T cells have been demonstrated effective in some solid tumor, while it is not good at treating tumor with low HLA level (61). For modularity, CARs are more friendly for all the elements of recognition and signal transduction are connected into one molecule, facilitating CAR insertion into other immune cells as macrophages and NK cells (62, 63). Unlike CARs, signal transduction of TCRs depends on the assembling with CD3 and co-stimulatory factors that limits the application of designed TCR in non-T cells (64).

CAR and TCR have different antigen affinities. Compared with TCR, CAR has a higher antigen binding affinity. At the nanomolar level, one of the directions for optimizing CAR-T cells is to increase the affinity of the extracellular binding domain scFv to achieve optimal targeting (65). CAR affinity can affect cytokine production, proliferation, and survival time, thereby affecting T cell function (66, 67). However, it is currently unclear whether anti-tumor ability is influenced by antigen affinity. Selected in thymus, all the TCRs on single positive T cells are able to bind to peptide MHC complex. TCR-T retains all the components of TCR signaling pathway that has higher antigen sensitivity which depends on the expression of MHC molecules on target cells (68, 69). Generally, there are three reasons affecting the antigen sensitivity of CAR and TCR: ① TCR and CAR form different immune synapses: TCR forms an ordered “target center” between T cells and target cells, while CAR cannot form stable immune synapses (70, 71). ② The number of ITAMs: TCR-CD3 complex contains a total of ten ITAMs involved in signal transduction, while CAR only contains three (72). ③ The degree of phosphorylation of signal molecules: Studies have shown that less phosphorylation of ITAM is induced by CD3 ζ in CAR, which further affects downstream signal transduction (73).

CAR-T and TCR-T are also distinct in manufacture. CAR-T cells are obtained from whole white blood cells that are activated *in vitro*, while TCR-T cells require antigen-specific T cells separated from peripheral blood mononuclear cell and following amplification by specific antigen peptides and cytokines (74, 75). It is vital for TCR-T to determine specific tumor related peptide. High resolution mass spectrometry is the most commonly used high-throughput method for directly identifying HLA-1 binding peptides in tumor cells (76, 77). The development of next-generation sequencing technologies helps us identify tumor specific antigens (also known as neoantigens) with higher specificity and fewer side effects (78, 79). Tumor specific T cells and TCRs were obtained through HLA-A polymerase staining (80), single-cell TCR sequencing (81), or antigen negative humanized mouse (82). The designed chains are expressed by viral transduction methods, such as adenoviruses, lentiviruses, and γ-retrovirus (83, 84). At present, some non-viral gene editing methods have been developed and applied in clinical trials, such as mRNA electroporation technology (85). For example, mRNA/RNA electroporation-based engineering has been evaluated in registered clinical trials of RNA-electroporated cMET-directed CAR-T cells (NCT03060356), RNA/mRNA-engineered anti-BCMA CAR-T cells, including Descartes-08 (NCT03448978 and NCT04146051), and mRNA-modified HBV-specific TCR-redirected T cells (NCT02719782, NCT04745403, and NCT04677088).

## 3 The clinical application of CAR-T and TCR-T

### 3.1 CAR-T and TCR-T for cancer treatment

CAR-T cell therapy has achieved unprecedented success in the treatment of hematological malignancies. Six CAR-T cells targeting CD19 and B cell maturation antigen (BCMA) were approved by FDA. Kymarih (Tisagenlecleucel) is the first approved CAR-T cell product targeting CD19 for three indications: ① Refractory or recurrent B-cell precursor acute lymphoblastic leukemia (B-ALL) (for patients under 25 years old) (86); ② Relapsed/refractory large b-cell lymphoma (R/R LBCL) after two or more systemic treatments (87); ③ New indications of R/R LBCL after at least two lines of therapy (88). As the second CAR-T cells approved by FDA, Yescarta (Axicabtagene Ciloreucel) is used to treat with refractory large B-cell lymphoma failed by conventional treatment via targeting CD19 (89). According to the results of latest phase III clinical trial, the estimated 4-year overall survival rate of the patients received Yescarta is higher than control (90). The clinical trial NCT03105336 showed that 74% patients with refractory non-Hodgkin’s lymphoma (NHL) achieved complete remission, which expanded the range of Yescarta indications (Figures 2A–C) (91). Tecartus (brexucabtagene autoleucel) was initially approved for the treatment of relapsed or refractory mantle cell lymphoma on the basis of the ZUMA-2 trial. (92). Its clinical application was subsequently extended to adults with relapsed or refractory B-cell acute lymphoblastic leukemia following the results of the phase II ZUMA-3 trial (NCT02614066). Among the 55 treated patients, 39 achieved complete remission or complete remission with incomplete hematologic recovery. (93). Figure 2D shows the duration of remission when patients undergoing subsequent allogeneic stem-cell transplantation were censored, whereas Figure 2E presents the corresponding analysis without censoring at transplantation. These complementary analyses illustrate the durability of response while also showing how subsequent transplantation may influence the interpretation of long-term remission. Figure 2F presents relapse-free survival, and Figure 2G shows overall survival after treatment. Taken together, these panels demonstrate that the clinical value of CAR-T therapy should be assessed not only by the initial remission rate but also by the durability of remission, the risk of relapse, and long-term survival (93). Figure 2H compares representative first-, second-, and third-generation anti-CD19 CAR architectures. First-generation CARs contain a CD3ζ signaling domain alone, whereas second-generation CARs incorporate an additional co-stimulatory domain, such as CD28 or 4-1BB. Third-generation CARs contain two co-stimulatory domains. This comparison provides the structural context for understanding currently approved anti-CD19 CAR-T products and highlights how receptor design may affect T-cell activation, expansion, and persistence. Breyanzi (lisocabtagene maraleucel; liso-cel) is an FDA-approved CD19-directed CAR-T product for adult patients with relapsed or refractory large B-cell lymphoma (41–45). As illustrated in Figure 2I, liso-cel uses a CD19-specific single-chain variable fragment, a transmembrane region, a 4-1BB co-stimulatory domain, and a CD3ζ signaling domain. The use of 4-1BB is intended to support T-cell expansion and persistence following antigen recognition (94, 95). Figure 2J summarizes the liso-cel manufacturing process, including leukapheresis, isolation of CD4^+^ and CD8^+^ T-cell subsets, activation and viral transduction, expansion, formulation, and administration of the final cellular product. Unlike CAR-T products with an undefined cellular composition, liso-cel is manufactured using separately expanded CD4^+^ and CD8^+^ CAR-T-cell components that are combined at a defined ratio. A single dose contains 50–110 × 10^6 CAR-positive viable T cells with approximately equal numbers of CD4^+^ and CD8^+^ cells (14, 95). This defined composition is intended to improve product consistency and illustrates the importance of manufacturing strategy, in addition to receptor design, in determining the characteristics of a clinical CAR-T product. Besides CD19, BCMA is also a good target for CAR-T because it expresses abnormally high in malignant plasma cells without expressing in normal tissues. Abecma (Idecabatagene Viclucel) is the first FDA approved CAR-T cell product targeting BCMA. The clinical trial (NCT03361748) showed that patients with recurrent/refractory myeloma 73% achieved remission and 33% achieved complete remission after treatment of Abecma (15). Carvykti (Ciltacabtagene Autoleucel) is the first approved Chinese CAR-T cell therapy against BCMA. Through two single domain VHHs, Carvykti increases the affinity for BCMA. In the latest follow-up results, deep and persistent responses were observed in both the standard and high-risk subgroups of patients receiving treatment at approximately 28 months of median follow-up (96). In addition to CD19 and BCMA, several other antigens have been explored as potential CAR-T targets for hematological malignancies, including CD20, CD22, CD7, CD30, CD38, SLAMF7/CS1, and CD99. Among these targets, several have already been evaluated in clinical or early-phase clinical studies, whereas others remain mainly at the preclinical or translational research stage. Representative registered clinical trials targeting different tumor antigens in hematological malignancies are summarized in Table 2.

**FIGURE 2:**
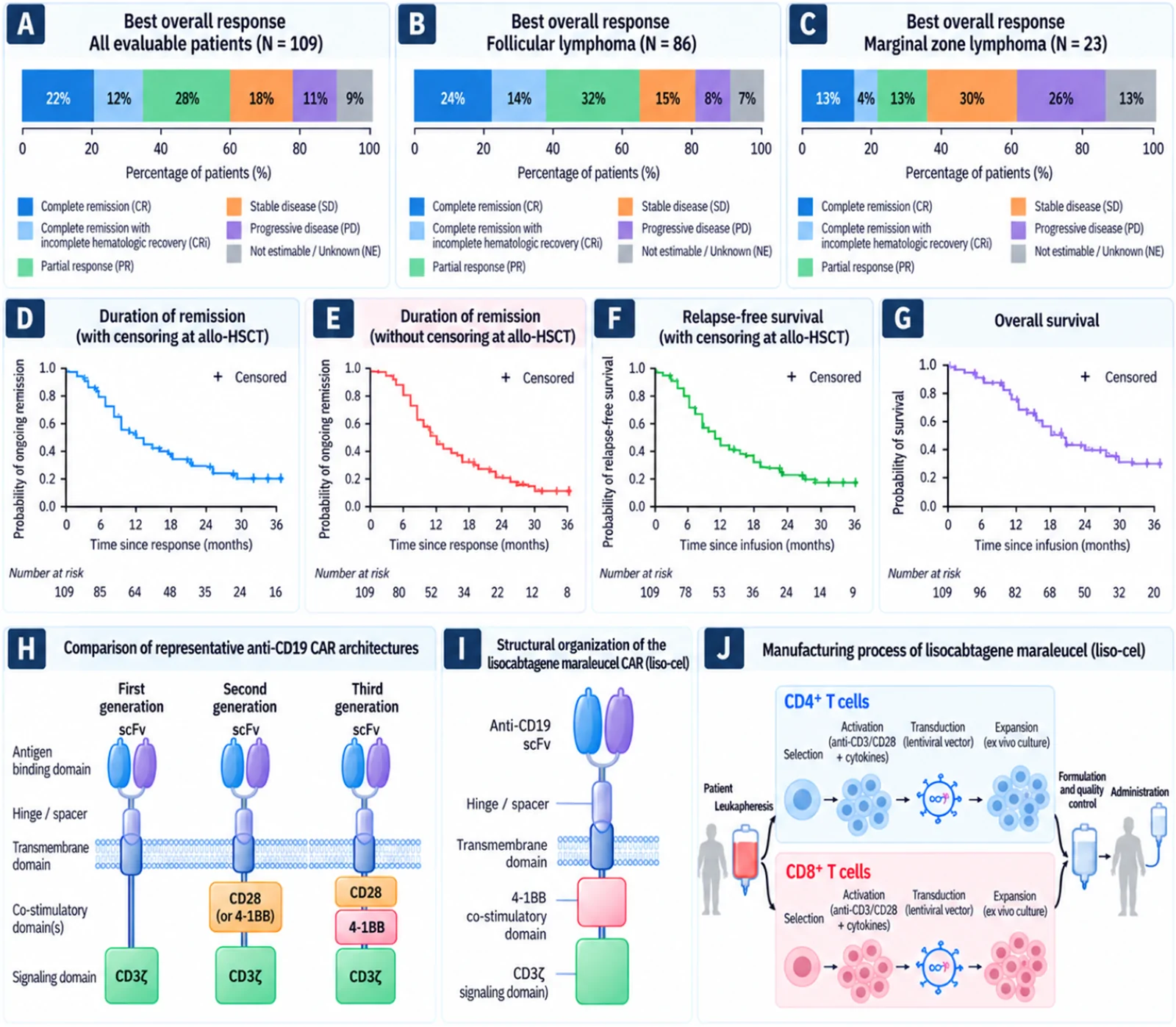
Clinical outcomes and representative design features of CAR-T-cell therapies for hematological malignancies. **(A–C)** Schematic summaries of the best overall responses in all evaluable patients, patients with follicular lymphoma, and patients with marginal zone lymphoma. Response categories are presented as original graphical summaries based on the findings reported in the cited studies and include complete remission (CR), complete remission with incomplete hematologic recovery (CRi), partial remission (PR), stable disease (SD), progressive disease (PD), and not estimable (NE), where applicable. Responses in the original studies were assessed by an independent radiology review committee. **(D–G)** Original schematic illustrations summarizing the reported clinical efficacy endpoints, including duration of remission with censoring at subsequent allogeneic stem-cell transplantation, duration of remission without censoring, relapse-free survival, and overall survival. These panels are conceptual summaries designed to illustrate the reported clinical outcomes and do not reproduce published Kaplan–Meier curves or underlying survival data. **(H)** Original comparison of representative first-, second-, and third-generation anti-CD19 CAR architectures. **(I)** Original schematic of the structural organization of the lisocabtagene maraleucel CAR, including the CD19-binding domain, hinge/spacer region, transmembrane domain, 4-1BB co-stimulatory domain, and CD3ζ signaling domain. **(J)** Original schematic overview of the lisocabtagene maraleucel manufacturing process, including leukapheresis, separate selection and activation of CD4^+^ and CD8^+^ T cells, transduction, expansion, formulation, and administration. This figure is an original schematic summary prepared for this review based on the studies already cited in the manuscript. It does not reproduce or adapt any previously published figures.

**TABLE 2:** Representative registered clinical trials of CAR-T therapy for hematological malignancies, grouped by target antigen.

Target antigen	Disease/indication	Clinical phase	Trial identifier	Study period	Main objective(s)
CD19	R/R HLM	I/II	NCT04790747	2021–2025	Safety and efficacy
	CD19-expressing malignancies	I	NCT04649112	2021–2023	Safety
	B-Cell lymphoma and B-ALL	II	NCT05281809	2022–2037	Feasibility
	R/R non-hodgkin lymphoma	I	NCT04545762	2020–2023	Safety and feasibility
	R/R MM	I	NCT04796688	2021–2024	Safety and efficacy
BCMA	R/R M/M	I/II	NCT04271644	2019–2024	Efficacy and safety
	High-risk NDMM	II	NCT05846737	2023–2028	Safety and efficacy
	R/R MM involving CRISPR-edited CAR-T cells	I	NCT05722418	2023–2027	Safety and best dose
	MM and AL amyloidosis	I	NCT04720313	2021–2026	Dose escalation and safety
	R/R plasma cell Blood Tumors	Early phase I	NCT06067581	2023–2025	Safety and efficacy
	High-risk SMM	II	NCT05767359	2023–2040	Safety and effectiveness
CD7	Hematologic malignancies	II	NCT05454241	2022–2025	Efficacy, safety and PK
	R/R CD7-positive T-ALL/lymphoma	I	NCT05212584	2022–2024	Safety and tolerability
	CD7-positive R/R T-ALL/LBL/AML	Early phase I	NCT05907603	2023–2025	Safety and pharmacokinetics
	R/R Lymphoid hematologic malignancies	I	NCT05170568	2021–2024	Safety and tolerability
GPRC5D	R/R MM or PCL	II	NCT05219721	2022–2024	Safety and efficacy
CD70	CD70 positive malignant hematologic	Early phase I	NCT04662294	2011–2027	Safety
IM21	R/R MM	Early phase I	NCT05478343	2021–2023	Safety and efficacy
CD28	Hematologic malignancies	Early phase I	NCT05239689	2022–2024	Safety and efficacy
CLL1	CLL1-positive hematological malignancies	Early phase I	NCT05252572	2022–2024	Safety and effectiveness
CS1	R/R MM	I	NCT03710421	2019–2023	Side effects and best dose
CD20	R/R B Cell non-hodgkin lymphomas	I/II	NCT03277729	2017–2037	Best dose and safety
CD138	R/R MM	I	NCT03672318	2029–2028	Efficacy
CD123	Hematologic and lymphatic malignancies	I	NCT04230265	2020–2025	Safety, side effects, harmlessness, therapeutical benefit
ROR1	Advanced hematologic and solid tumor malignancies	I	NCT05694364	2023–2026	Dose escalation/Dose expansion study
NKG2D	R/R AML/MDS	I	NCT04167696	2019–2035	Recommended dose

Abbreviations: R/R, relapsed/refractory; B-ALL, B-cell acute lymphoblastic leukemia; MM, multiple myeloma; NDMM, newly diagnosed multiple myeloma; SMM, smoldering multiple myeloma; PCL, plasma cell leukemia; T-ALL, T-cell acute lymphoblastic leukemia; LBL, lymphoblastic lymphoma; AML, acute myeloid leukemia; MDS, myelodysplastic syndrome.

In treatment of solid tumor, the therapeutic effects of CAR-T cells are influenced by different microenvironment compared to hematological malignancies. Generally, there are some obstacles that need to overcome: ① Absence of effective surface antigens; ② Tumor heterogeneity; ③ Deficiency of T cells infiltration; ④ Immunosuppressive microenvironment. At present, there are no approved CAR-T cell products targeting solid tumor antigens, but many clinical studies have been published or are currently underway.

The clinical trial (NCT01869166) evaluated the safety and effectiveness of CAR-T cells targeted epidermal growth factor receptor (EGFR) in the patients of metastatic pancreatic cancer (PCA). Four patients achieved partial remission within 2–4 months, and eight patients were in a stable condition within 3–4 months in all 16 patients enrolled. From the first cycle of CAR-T cell therapy, the median progression free survival period was 3–4 months and the median total survival period of nine assessable patients was 4.9 months with fever/fatigue. Adverse events such as nausea/vomiting, mucosal/skin toxicity, pleural effusion, and pulmonary interstitial exudation were reversible. Representative clinical findings of EGFR-targeted CAR-T therapy are schematically summarized in Figure 3A. The reported study demonstrated reduction of the primary pancreatic tumor and regression of metastatic liver lesions in responding patients (97). These findings support the potential clinical activity of EGFR-targeted CAR-T therapy in metastatic pancreatic cancer (97). In addition to EGFR, mesothelin is also considered a potential target for CAR-T therapy in pancreatic cancer. Although early-phase clinical studies showed limited clinical efficacy, mesothelin-targeted CAR-T cells demonstrated acceptable safety and detectable persistence in peripheral blood. Figure 3B conceptually summarizes the post-infusion expansion, peak abundance, and subsequent persistence of mesothelin-targeted CAR-T cells reported in early-phase studies (98). Human epidermal growth factor receptor 2 (HER-2) is generally used as a targeted antigen for CAR-T cell therapy in various tumors, such as sarcoma, high-grade glioma and colorectal cancer. In a clinical study of phase I/II therapy with dose escalation for metastatic or recurrent sarcoma, CAR-T cells showed no dose limiting toxicity. Among 17 evaluable patients, four were in stable condition within 12 weeks–13 months, and one of the three patients who underwent tumor resection achieved 90% necrosis of tumor tissue (99). CAR-T cells also have no dose-limited toxic effects on HER-2 positive glioblastoma patients. In the first phase clinical trial, CAR-T cells were still detected in peripheral blood after 2 months of infusion, with eight patients remaining stable for more than 8 months and eight patients still progressing (100). According to the results of the first phase trial, it is necessary to evaluate the efficacy of HER2-CAR-T cells as a single drug for glioblastoma or in combination with other immunomodulatory methods in the second phase of the study. Although CAR-T has shown significant inhibition against sarcomas and high-grade gliomas, it has some serious fatal side effects in colon cancer patients with lung and liver metastases. After cell infusion, patients developed respiratory distress due to obvious lung infiltration and died 101 days after treatment (101). The same effects of on-target/off-tumor toxicity also occurred in clinical trials of CAR-T targeting epidermal growth factor III (EGFRvIII) for the treatment of recurrent glioblastoma. Two patients experienced severe hypoxia, and even one patient died due to treatment-related side effects after the highest dose was administered (102). IL-13Rα2 is associated with reduced survival rate of gliomas and considered as target for CAR-T treatment of gliomas. In a case of a 50 years old male patient with right temporal lobe glioblastoma, intracavitary administration can prevent local tumor recurrence but cannot effectively control distant tumor progression. After intraventricular infusion, all intracranial and spinal tumors decreased, while the patient recurred less than a year after the disease subsided. Preliminary assessment of the correlation between downregulation of target antigens and recurrence (103). The immune escape caused by decreased expression of target antigens is another major obstacle that needs to be overcome in CAR-T cell therapy. α- Folate receptor (FR-α) is a tumor associated antigen of ovarian tumors, and CAR-T with scFv against FR-α exhibited inhibition to tumor progression in mouse models, but limited effects were observed in clinical trials, and CAR-T cells cannot maintain in patients for a long time (104). GD2 is a validated immunotherapy target for recurrent or refractory neuroblastoma (NB). In the first phase clinical results of third-generation CAR-T cell products targeting GD2, combination with cyclophosphamide and fludarabine (Cy/Flu) can promote CAR-T cell expansion, suggesting that the anti-tumor effects of various independent methods can be promoted through the combination of multiple regimens (105). Due to various limiting factors, CAR-T therapy has not shown significant efficacy in the treatment of solid tumors. The ongoing clinical studies on CAR-T targeting solid tumors are summarized in Table 3.

**FIGURE 3:**
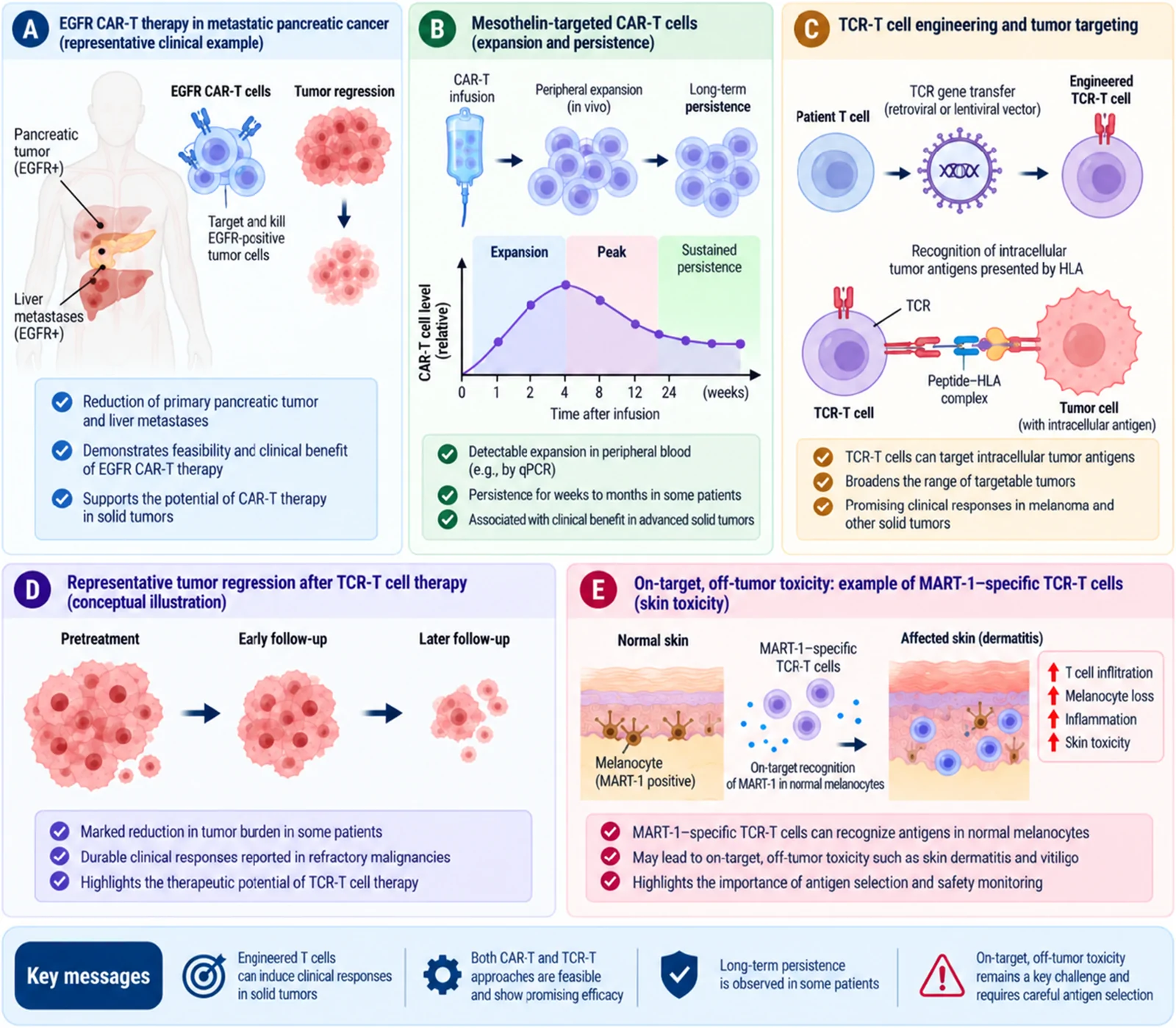
Representative clinical applications and biological features of engineered T-cell therapies. **(A)** Schematic summary of reported clinical responses to EGFR-targeted CAR-T therapy in metastatic pancreatic cancer, including reduction of primary and metastatic tumor burden. **(B)** Conceptual illustration of the expansion, peak abundance, and persistence of mesothelin-targeted CAR-T cells following infusion. **(C)** Schematic overview of TCR-T-cell engineering and recognition of intracellular tumor antigens presented by peptide-HLA complexes. **(D)** Conceptual representation of tumor-burden reduction following TCR-T-cell therapy. **(E)** Schematic illustration of on-target, off-tumor toxicity associated with MART-1-specific TCR-T cells, showing immune-mediated damage to normal melanocyte-containing skin. This figure is an original schematic illustration independently created by the authors to summarize information from the cited literature. No previously published figure or graphical element was directly reproduced.

**TABLE 3:** Representative clinical trials of CAR-T targeting solid tumors.

Antigen	Tumor type	Phase	NCT number	Time	Main objective(s)
CEA	CEA-positive Advanced/​Metastatic malignant solid tumors	I	NCT06010862	2023–2026	Safety and tolerability
CD70	CD70-positive Advanced/​Metastatic solid tumors	I	NCT06010875	2023–2026	Safety and efficacy
EpCAM	Advanced solid tumors	I	NCT02915445	2026–2023	Safety and efficacy
MUC1-C	Advanced or metastatic epithelial derived solid tumors	I	NCT05239143	2022–2039	Safety, tolerability and response
ROR1	ROR1+ R/R TNBC or NSCLC	I	NCT05274451	2022–2026	Safety and tolerability
HER-2	HER2 positive solid tumors	I	NCT03740256	2020–2038	Safety and efficacy
CLDN6	CLDN6-positive relapsed or refractory advanced solid tumors	I/II	NCT04503278	2020–2037	Safety and efficacy
B7-H3	Pediatric patients With R/R B7-H3+ solid tumors	I	NCT04897321	2022–2027	Safety and MTD
GD2	High risk and/​or R/R neuroblastoma or other GD2-positive solid tumors	I/II	NCT03373097	2018–2027	Safety and efficacy
CLDN18.2(CT041)	CLDN18.2 expression-positive, advanced gastric/esophagogastric conjugate adenocarcinoma	I/II	NCT04581473	2020–2038	Efficacy, safety and pharmacokinetics
CLDN18.2/Cytokines	Advanced solid tumors	I	NCT05393986	2022–2024	Safety, tolerability, preliminary efficacy and pharmacokinetics
GPC3	GPC3-positive solid tumors	I/II	NCT05120271	2022–2039	Safety and RP2D
MSLN(UCLMM802)	Mesothelin-positive advanced malignant solid tumors	I	NCT05848999	2023–2025	Safety, tolerability and preliminary efficacy
NKG2D	R/R NKG2DL + solid tumors	I	NCT05131763	2021–2023	Safety and clinical activity
HLA-G (IVS3001)	Advanced HLA-G-Positive solid tumors	I/II	NCT05672459	2023–2029	Safety, tolerability, pharmacokinetics and clinical activity
EGFR806	Recurrent/​Refractory solid tumors	I	NCT03618381	2019–2038	Safety, feasibility, and efficacy
B7-H3 (TAA06)	TAA06-positive advanced solid tumor	I	NCT05190185	2021–2023	Safety, tolerability, distribution, proliferation and persistence
IL-15 armored GPC3-specific CAR	Pediatric solid tumors	I	NCT04377932	2021–2040	Maximum tolerated dose, side effect, peisistance
TGFβR-KO CAR-EGFR	Advanced EGFR-positive solid tumors	I	NCT04976218	2022–2024	Anti-tumor activities and safety profiles
Anti-PD-L1/Anti-CD22	Solid tumors	I	NCT04556669	2020–2025	Adverse reaction, pharmacokinetics, and the outcomes
VEGFR1 and PD-L1	Pleural or peritoneal metastases	I	NCT05477927	2022–2024	Safety and effectiveness
GD2/PSMA	GD2 and PSMA positive tumor	I/II	NCT05437315	2022–2026	Feasibility, safety and efficacy, persistency
CD40/MSLN	MSLN positive Advanced/​Metastatic solid tumors	I/II	NCT05693844	2023–2025	Dose limiting toxicities, maximum tolerated dose

Despite substantial advances in CAR-T engineering, clinical efficacy in solid tumors remains considerably lower than that observed in hematological malignancies. Although numerous engineering strategies—including dual-target CARs, armored CAR-T cells, chemokine receptor modification, and local delivery approaches—have demonstrated encouraging preclinical activity, their clinical translation has been relatively limited. This discrepancy suggests that no single engineering strategy is sufficient to overcome the multifactorial barriers presented by solid tumors, including antigen heterogeneity, physical stromal barriers, immunosuppressive tumor microenvironment, and T-cell dysfunction. Therefore, future therapeutic success will likely depend on combinatorial engineering approaches integrating optimized antigen selection, metabolic reprogramming, immune checkpoint modulation, and microenvironment remodeling rather than relying on individual modifications alone.

TCR-T has become an emerging cellular immunotherapy for the treatment of solid tumors due to its lack of restriction by surface antigens. The clinical efficacy of TCR-T therapy in treating solid tumors has been validated in many preclinical studies and clinical trials worldwide. At present, there are two kinds of target antigens of TCR-T considered in clinical practice: tumor associated antigen (TAA) and tumor specific antigen (TSA) (106). The ideal target antigen is tumor specific. With the development of bioinformatics and sequencing technology, new approaches for identifying new antigens have been explored, and the target antigen library of TCR-T has also been expanded. In 2006, the first clinical trial of TCR-T targeting the melanoma antigen MART-1 was reported and showed significant regression of metastatic tumors in patients receiving TCR-T treatment (107). Figure 3C schematically illustrates the general process of TCR gene transfer and tumor-antigen recognition by engineered T cells. Representative clinical responses to TCR-T therapy are conceptually summarized in Figure 3D. Reported studies demonstrated substantial reductions in metastatic tumor burden in responding patients (107). These observations provided important proof-of-concept for the clinical activity of TCR-engineered T cells in solid tumors (107). Since that, there have been over a hundred clinical trials of TCR-T in solid tumors such as melanoma, synovial sarcoma, rectal cancer, and myeloma, providing detailed reference data for the application and development of TCR-T in solid tumors. Melanoma is one of the most malignant tumors in skin cancer. Once it metastasizes, it is not sensitive to postoperative adjuvant radiotherapy and chemotherapy, and the prognosis is extremely poor. At present, TCR-T cell immunotherapy has the most clinical research in melanoma. The results of clinical study targeting MART-1 also provide a basis for its clinical promotion. This study used MART-1 targeted TCR-T cells and MART-1 peptide pulsed DC vaccines. Among the 14 HLA-A2 melanoma patients enrolled, more than half (69%) showed significant tumor regression, but its specificity still needs further improvement (108). Although targeting TAA have shown good therapeutic effect in CAR-T treatment, some severe adverse side effects occur in some cases. However, enhanced antigen recognition may also increase the risk of immune-related toxicity. Figure 3E schematically illustrates on-target, off-tumor toxicity associated with MART-1-specific TCR-T cells. Because MART-1 is also expressed by normal melanocytes, antigen-specific T-cell activation can result in immune-mediated damage to normal melanocyte-containing tissues and contribute to treatment-associated dermatitis. In a recent phase I/IIa study targeting MART-1, treatment was terminated prematurely because of severe dose-dependent toxicity involving the skin, eyes, and ears (109). There is a close relationship between the affinity of TCR and therapeutic efficacy, but the results of a clinical trial suggest that a higher affinity for TCR may cause reduced safety. Johnson et al. found TCR-T with TCR recognizing MART-1 melanoma antigen derived from TIL clones of excised melanoma lesions showed significant regression of metastatic melanoma, while TCR-T cells with an affinity enhanced TCR caused extensive erythematous rash, eye and ear toxicity in most patients (110). The clinical results of another tumor associated antigen gp100 also showed similar adverse events, but these events did not lead to trial termination, and the overall survival rate of the treatment group was 14% higher than that of the control group (73% vs. 59%) (111). NY-ESO-1 is another target of TCR-T for melanoma and synovial sarcoma treatment. In a recent clinical trial, the TCR-T with affinity enhanced NY-ESO-1 specific TCR and endogenous TCR silencing mediated by siRNA showed significant anti-tumor effects and early onset CRS in tumor patients with high levels of NY-ESO-1 expression, and its safety met clinical applicability standards (112). The clinical results of targeted MAGE-A3 treatment for melanoma, synovial sarcoma, and esophageal cancer were not ideal, with some patients experiencing severe neurological toxicity, shortness of breath, pulmonary edema, and death (113). MAGE-A4 showed no significant effect in clinical trials of esophageal cancer patients, but significant relief was observed in sarcoma patients (114). MAGE-A10 also serves as a targeted antigen for TCR-T. ADP-A2M10 expresses high affinity MAGE-A10 specific TCR and targets MAGE-A10 tumor cells. Clinical trials targeting non-small cell lung cancer, advanced head and neck squamous cell carcinoma, melanoma, or urothelial carcinoma in phase I have not shown significant results, and have all reported adverse events such as lymphopenia, neutropenia, leukopenia, and thrombocytopenia (115, 116). TSAs are ideal target antigen, as they are not expressed in normal tissues and rarely experiences adverse events. TSAs in clinical practice are mainly mutation related neoantigens and viral antigens. The clinical efficacy of public new antigens such as TP53, KRAS, PIK3CA, etc., has been reported (117–119). It is worth mentioning the cancer-related viral antigens induced by viral infections, such as the E6 and E7 proteins of human papillomavirus. Partial clinical reactions of TCR-T targeting both HPV16 E6 and HPV16 E7 proteins were reported without significant toxicity (120, 121). Among these challenges, tumor heterogeneity represents one of the most important factors limiting the efficacy and durability of adoptive T-cell therapy. Unlike hematological malignancies, in which lineage-specific antigens such as CD19 and BCMA can be relatively consistently expressed, solid tumors exhibit substantial heterogeneity in antigen expression, genetic characteristics, spatial distribution, and interaction with the surrounding microenvironment. This diversity creates significant obstacles for effective tumor recognition and complete tumor elimination by engineered T cells.

Antigen heterogeneity may result in incomplete targeting because only a subset of tumor cells may express sufficient levels of the target antigen required for CAR-T cell recognition. Following selective elimination of antigen-positive tumor cells, antigen-low or antigen-negative clones may survive and contribute to disease relapse. In addition, spatial heterogeneity within tumors can generate distinct regions with different levels of oxygen, nutrients, stromal components, and immune suppressive factors, further affecting T-cell infiltration, activation, and persistence.

The heterogeneous tumor microenvironment represents another major limitation for adoptive T-cell therapy. Differences in immune-cell composition, inhibitory cytokine expression, metabolic conditions, and checkpoint signaling among tumor regions may impair T-cell expansion and effector function. Therefore, overcoming tumor heterogeneity requires integrated strategies, including improved antigen selection, multi-antigen targeting, optimized CAR design, enhanced resistance to immunosuppressive signals, and rational combination therapies. The current TCR-T clinical trials are summarized in Table 4. Collectively, the current clinical development and emerging applications of adoptive T-cell therapy across different cancer types are summarized in Figure 4.

**TABLE 4:** Table 4 Registered TCR-T clinical trials targeting solid tumors.

Antigen	Tumor type	HLA	Phase	NCT number	Time	Goals
NY-ESO-1	Advanced solid tumors	HLA-A2+	I	NCT05881525	2023–2025	Safety and efficacy
NY-ESO-1 (TAEST16001)	Soft tissue sarcoma	HLA-A * 02:01	I	NCT04318964	2020–2024	Safety, tolerance, PK, PD characteristics, and preliminary efficacy
NY-ESO-1/dnTGFbetaRII	Advanced malignancies	HLA-A2.1	I/II	NCT02650986	2017–2032	Safety and feasibility, T cell differentiation that correlates with higher anti-tumor responses
MAGE-A4/8	Solid tumors	HLA-A*02:01	I	NCT03247309	2018–2024	Safety
MAGE-A1 (TK8001)	Advanced-stage/​Metastatic MAGE-A1+ solid tumors	HLA-A*02:01	I/II	NCT05430555	2022–2037	Safety, tolerability and anti-tumoral activity
MAGE-A3/​A6	Adults with advanced cancers	HLA-DPB1*04:01	I	NCT03139370	2017–2023	Safety and efficacy
KK-LC-1	KK-LC-1 positive epithelial cancers	HLA-A01:01	I	NCT05035407	2022–2026	Safety of different doses of KK-LC-1 TCR T cells plus aldesleukin
KK-LC-1 or neoantigens	Advanced lung cancer or other solid tumors	MHC-A11	I	NCT03778814	2018–2033	Safety, tolerance and preliminary efficacy
KRAS G12V	Advanced or metastatic solid tumor	HLA-A*11:01	I/II	NCT06105021	2023–2029	Safety, tolerability, OBD and RP2D
TP53 R175H mutation	TP53 R175H mutant solid tumors	HLA-A*02:01	I	NCT05877599	2023–2039	Safety and preliminary anti-tumor activity
KRAS/TP53/EGFR	Relapsed/refractory solid tumors	HLA-A/HLA-C/HLA-DRB	I/II	NCT05194735	2022–2029	DLT and MDT or RP2D
MAGE-A1, HPV16 E7, PRAME	Locally advanced (unresectable) or metastatic solid tumors	HLA-A*02:01, HLA-C*07:02	I	NCT05973487	2023–2026	Safety, preliminary efficacy of single and repeat dose regimens of TCR'Ts as monotherapies and as T-Plex combinations
IMA203/IMA203CD8	Recurrent and/​or refractory solid tumors	HLA-dextramer	I	NCT03686124	2019–2028	Safety and tolerability
NY-ESO-1 and/​or LAGE-1a	Advanced solid tumors	HLA-A*02	I	NCT04526509	2020–2025	Safety and RP2D
PRAME	Metastatic/unresectable tumors	HLA-A2	I/II	NCT04262466	2020–2026	Safety and efficacy
NY-ESO-1 and/​or LAGE-1a	Solid tumors	HLA-A*02	II	NCT03967223	2019–2026	Safety and efficacy

**FIGURE 4:**
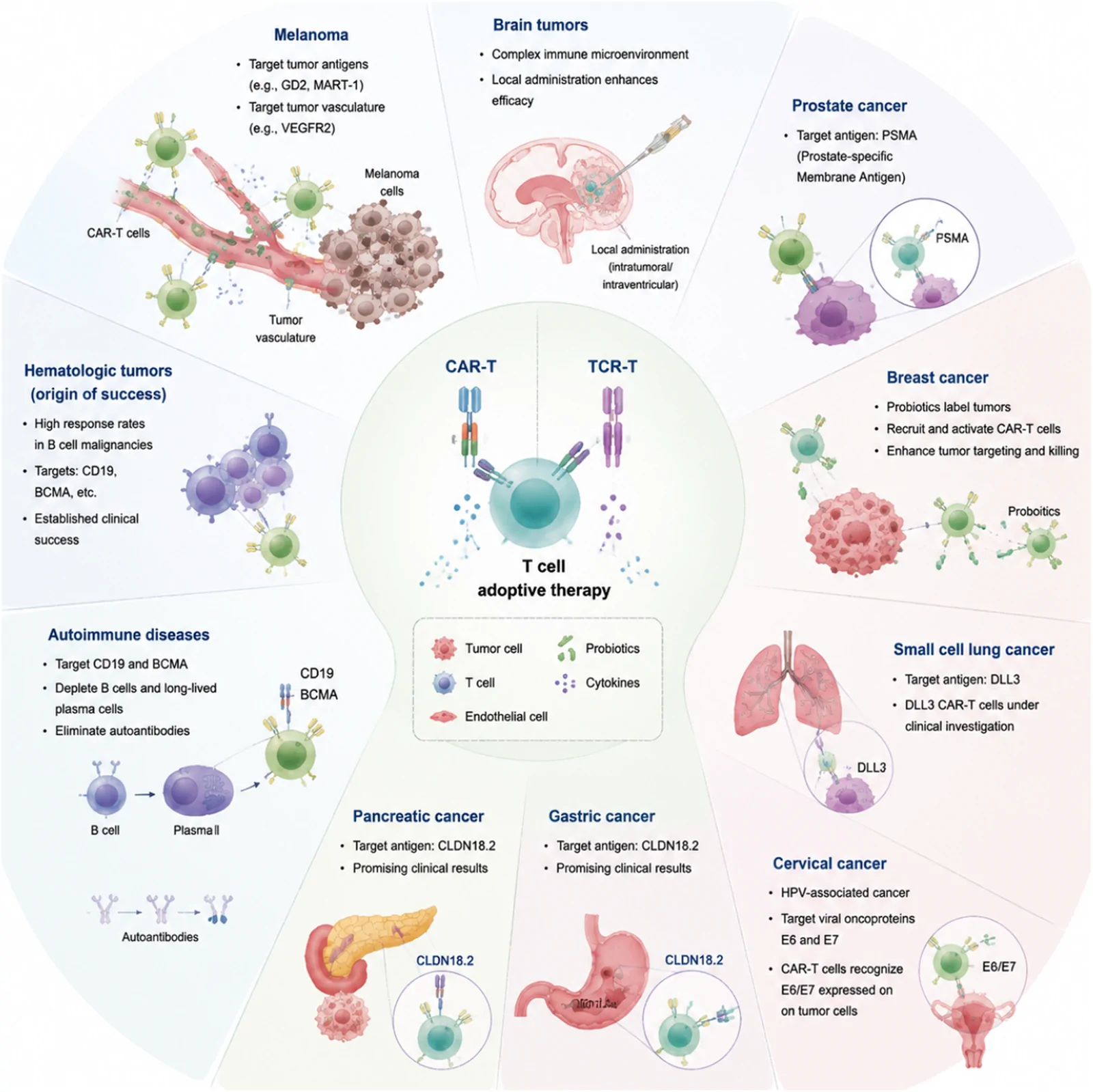
The cutting-edge applications of T-cell adoptive therapy across different cancer types. CAR-T cell therapy was initially successful in hematologic tumors, and its therapeutic effect in a variety of solid tumors has been gradually explored. In melanoma, in addition to targeting antigens on tumor cells, attacking the vasculature with CAR-T can also inhibit melanoma. Brain tumors have a more complex immune environment than other tissues, and local administration strategies can promote the effectiveness of T cell therapy in brain tumors. In prostate cancer, the target antigen is mainly PSMA, which is a prostate specific membrane antigen. In breast cancer, probiotics are used to label tumors and direct CAR T cells to target them. DLL3 is an emerging target for small cell lung cancer, and T-cell adoptive therapies targeting DLL3 are under clinical investigation. Cervical cancer is a cancer explicitly caused by HPV virus infection, so CAR-T therapy has been used to target HPV cancer proteins E6 and E7. Both pancreatic cancer and gastric cancer belong to digestive system malignancies, and CLND18.2 is their common tumor antigen, which has shown good results in clinical trials. Since T cells and B cells are highly correlated with autoimmune diseases, the application of immune cell therapy in autoimmune diseases is gradually developing. CD19 and BCMA double-targeted CAR-T can not only induce B cell depletion, but also target long-life plasma cells, which has the potential to completely remove autoantibodies to achieve better clinical results. This figure is an original schematic illustration created by the authors based on information reported in the cited literature. No previously published figure was reproduced or adapted.

### 3.2 CAR-T and TCR-T for autoimmune diseases treatment

Autoimmune diseases are caused by the deposition of autoantigens and immune complexes resulting in autoimmune dysfunction. The reasons for immune tolerance failure are firstly due to the presence of autoantibody and autoreactive lymphocytes. At present, there is no specific treatment for autoimmune diseases. Immunosuppressive drugs such as methotrexate and cyclosporine are often used to inhibit immune responses to reduce pain and inflammation. However, it does not target the origin of the autoimmune diseases, which may increase the risk of infection and malignant cancer in patients. The immunotherapies based on monoclonal antibodies have shown positive effects in the treatment of autoimmune diseases. Rituximab, which chimeras with human CD20 specific antibodies, has been shown to have an inhibitory effect on progressive joint injury and improve clinical outcomes within 1 year in clinical trials (122). Besides, rituximab can also improve interstitial lung disease and skin fibrosis associated with skin fibrosis and systemic sclerosis (SSC) (123, 124). Currently, rituximab is the standard clinical treatment for immune-mediated necrotizing myopathy (IMNM) and pemphigus vulgaris (PV) (125, 126). Belimumab is a recombinant human IgG-1 λ monoclonal antibodies against B cell activating factor (BAFF). It has been approved for use in active autoantibody positive systemic lupus erythematosus (SLE) patients aged 5 years or older and was observed to be effective in patients with active lupus nephritis (127). Based on these clinical research foundations, CAR-T cells with monoclonal antibody functional regions as extracellular binding domains can be applied to eliminate autoreactive immunocytes in autoimmune and immune-mediated diseases. In SLE, CAR-T cells have been applied to deplete abnormally activated B cells (128). In (NZB × NZW) F+1(NZB/W) lupus model, CAR-T cells prepared by A-MLV retrovirus and VSV-G virus transduction both showed depletion of CD19 B cells in 7-month-old female mice. Subsequently, a significant impact of CD19 targeted CAR on disease occurrence was observed in the MRL fas/fas (MRL lpr) mouse model (129). Further studies have found that CD19 CAR-T cell therapy can also prevent SLE effects, delay the onset of disease and improve symptoms in MRL lpr mouse models (128). Based on results of animal model, clinical trials targeting CD19 CAR-T cells have been launched. In a clinical trial, five refractory SLE patients (resistant to several immunosuppressive drugs) showed improved clinical symptoms with depletion of B cells after CAR-T infusion prepared with autologous T cells, and all patients met DORIS standard in terms of remission (130). Another trial showed that CAR-T cells targeting CD19 realized the remission of SLE of all four patients who resisted to steroids, hydroxychloroquine, mycophenolate ester, cyclophosphamide, immunoglobulin, rituximab, and belimumab (131). CD19 targeted CAR-T cell therapy was also observed to have a rapid relief effect in a 20-year-old female patient with severe refractory SLE. The patient did not experience any adverse reactions and double stranded DNA autoantibody levels rapidly decreased to negative levels within 5 weeks after CAR-T cell administration (132). In addition to CD19, B cell maturation antigens (BCMA), CD20, and others are also targeted antigens for CAR-T immunotherapy for autoimmune diseases. BCMA is only expressed on plasma cells and plasma cells, and it is also a legendary target for the treatment of recurrent/refractory multiple myeloma. In the later stage of the disease, it is ineffective for long-lived plasma cells with CD19 targeting CAR-T cells, which may still accumulate and lead to sustained production of autoantibodies. Therefore, dual targeting of CD19 and BCMA has the potential to achieve better clinical outcomes by completely removing autoantibodies. In a phase I clinical trial (NCT04162353), SLE symptoms and stage IV diffuse large B-cell lymphoma were relieved in a 41 year old SLE patient received dual targeted CAR-T treatment (133). Besides SLE, CAR-T have been proven effective in other autoimmune diseases. Clinical studies evaluated the safety and efficacy of CD19-CAR-T cell therapy in patients with severe anti synthetase syndrome. The subjects reported active myositis, interstitial lung disease, muscle pain, and grade IV muscle weakness. After receiving treatment, the patient’s symptoms were alleviated (134). CAR-T cell therapy against BCMA has also been used to treat recurrent or refractory AQP4 IgG serum positive optic neuritis spectrum disorders. All 12 enrolled patients achieved improvements in quality of life and other aspects, with 11 patients showing no recurrence and a decreasing trend in AQP-4 antibodies (135). Clinical research on CD19/BCMA chimeric CAR-T cells for refractory Sjogren’s syndrome patients, autoimmune hemolytic anemia, and vasculitis is currently being recruited (NCT05085431, NCT05263817). Table 5 summarized the clinical trials of CAR-T treating autoimmune diseases.

**TABLE 5:** Clinical trial of CAR-T cells for autoimmune diseaseable.

CAR-antigen	Condition	Phase	NCT number	Time	Goals
CD19	RSSc, R SS, RDM, refractory the antineutrophil cytoplasmic antibody-associated vasculitides	I	NCT06056921	2023–2026	Efficacy and safety
	R/M S SLE	I/II	NCT06106906	2023–2026	Safety and tolerability
	Refractory/​Moderate-to-severe SLE	I/II	NCT06106893	2023–2026	Safety and Efficacy ((CAR-γδT cells))
CD19	E/R AD	Unknow	NCT05859997	2023–2025	Safety and efficacy
	Moderate to severe active SLE	I	NCT05765006 (Relma-cel)	2023–2025	Safety tolerability pharmacokinetics and pharmacodynamics of relma-cel
	RLN	I	NCT05938725	2023–2026	Incidence adverse events (AEs) and laboratory abnormalities; frequency of dose limiting toxicities at each dose level
	SLE or AIT	Early phase 1	NCT05930314 (CNCT19)	2023–2025	Ate the safety and preliminary efficacy
	S/R SLE	I/II	NCT05798117 (YTB323)	2023–2026	Safety, efficacy and cellular kinetics
	ASLE	I/II	NCT06121297 (CABA-201)	2023–2027	Safety and efficacy
BCMA (Descartes-08)	SLE	II	NCT06038474	2023–2026	Safety and tolerability
BCMA/CD19	R/​R SLE	I	NCT05474885	2022–2025	Safety and tolerability
CD19-BCMA	RSLE	I	NCT05846347 (GC012F)	2023–2025	The recommended phase II dose of GC012F injectioni
BCMA (NXC-201)	ALA	1b	NCT06097832	2024–2039	Safety and efficacy
BCMA	NMOSD,MG,CIDP,IMNM	Early phase 1	NCT04561557	2020–2024	Safety and efficacy
CD7	CD UC Dermatomyositis Still disease	Early phase 1	NCT05239702	2022–2024	Safety and effectiveness
CD19/BCMA	R POEMS, SAmyloidosis, AIHA, and vasculitis	Early phase 1	NCT05263817	2021–2024	Safety and effectiveness
FKC288	R/RS ALA	I	NCT05978661	2023–2026	Evaluate the safety, explore the recommended phase II dose
CD19/BCMA	RSS	Early phase 1	NCT05085431	2023–2024	Safety and tolerability
CD19/BCMA	Refractory scleroderma	Early phase 1	NCT05085444	2021–2024	Safety and effectiveness
CD19/CD20	R/R AQP4-IgG seropositive NMOSD	I	NCT03605238	2018	Safety and efficacy

CAR Treg and chimeric autoantibody receptor T cell (CAAR-T) therapy are new therapies for treating autoimmune diseases (136). Treg cell therapy has shown efficacy in preventing and treating autoimmune diseases caused by Treg cell abnormalities in animal models (137). CAR Treg cells can induce cell apoptosis by binding to Fas ligand (FasL) on effector T cells or competitively inhibit effector T cell proliferation by binding to IL-2 (138, 139). Unlike CAR-T cells that search for target antigens on target cells through extracellular antibody domains, CAAR loads autoantigen fragments onto T cells to target autoantibodies on antigen-specific B cells, thereby specifically killing B cells that produce autoantibodies without affecting normal B cells (140). DSG3 specific CAAR-T has shown considerable specificity and safety in preclinical studies, and its clinical efficacy in patients with mucosal dominant PV is currently being evaluated (141, 142). The cutting-edge research of T cell adoptive therapy in different cancer species is summarized in Figure 4.

## 4 Limitations and solutions of T cell adoptive therapy

Cell therapies have shown great promise in the treatment of cancer, and CAR-T cell therapies in particular have achieved excellent contributions in the treatment of malignant hematological tumors. However, these therapies still have certain limitations that make their clinical application face a series of challenges, such as potentially life-threatening toxicity, immune escape, tumor heterogeneity, immune-suppressive microenvironments, tumor accessibility, and tumor infiltration.

### 4.1 Common toxic effects and ameliorative strategies

Toxic effects induced by cellular immunotherapy can be broadly categorized into three types: (1) Cytokine release syndrome (CRS) due to overactivation of T cells and high levels of cytokine release. CRS is the most common and serious adverse event, which has not been observed in preclinical models but was first identified in clinical studies (143). (2) Off-target toxicity (caused by engineered T cells recognizing non-target antigens with cross-reactivity) and targeted non-target toxicity (caused by engineered T cells recognizing target antigens expressed on normal tissues). (3) The immune effector cell-associated toxicity syndrome (ICANS). The associated neurotoxicity due to the interaction of T cells with other immune effector cells, which usually presents with self-limiting symptoms such as headaches, encephalopathy, tremors, and seizures, but deaths have been reported as a result of it (144).

Currently, the improvement of CAR-T and TCR-T cells is mainly aimed at enhancing their tumor specificity and thus reducing the associated toxic effects. Tumor specificity needs to be considered from the selection of target antigens. The two target antigens considered clinically are tumor-specific antigens and tumor-associated antigens, the optimal choice is tumor-specific antigens that are expressed only in tumor cells. However, the number of safe and effective specific antigens identified to date is very limited, so that the development and application of neoantigens has become attractive (145). The second strategy to address the safety issue is to modify the structure of CAR or TCR. CAR-T cells are activated by binding to antigenic epitopes on the surface of target cells. There exists a threshold value for this activation, below which no effective cytotoxic effect can be produced, while above which cytokines associated with adverse reactions and abnormal activation of the immune system are appeared. The modular structure of CARs can be optimized to reduce toxicity. Selection of antigen-binding domains with appropriate affinity and optimization of hinge regions and transmembrane regions may be effective in reducing toxicity (146). In a phase I clinical trial, a representative CD19-BBz variants was created by a tertiary structure prediction program (Phrye2). The prototype CD19-BBz CAR structure was modified using genetic engineering techniques to replace the sequences encoding both extracellular and intracellular domains of CD8α molecules. T cells transduced with the CD19-BBz (86) variant CAR vectors produced significantly lower levels of cytokines while retaining potent cytotoxicity against CD19-positive tumor cells both *in vitro* and *in vivo*. Six of 11 patients achieved complete remission, and no neurotoxicity or CRS was observed. These findings illustrate how rational CAR structural optimization may improve the balance between antitumor activity and treatment-related toxicity, as schematically summarized in Figures 5A,B. Another option to modify the CAR structure is to utilize control switches which can control CAR expression or Logic-Gated CARs (147). The off-target toxicity caused by TCR may be caused by the self-reaction or cross-reaction between the α and β chains of T cells and the exogenous α and β chains (148). Knocking out endogenous TCR may reduce the adverse effects of TCR-T cell immunotherapy (149). Retroviral vectors and siRNAs have been used to silence the expression of endogenous TCR genes, thereby enhancing the expression of transduced tumor-specific TCRs while minimizing potential TCR mismatches (112). Although substantial progress has been achieved in reducing treatment-related toxicity through receptor engineering, affinity optimization, logic-gated CARs, and safety switches, these strategies often compromise antitumor potency or increase manufacturing complexity. More importantly, the balance between therapeutic efficacy and safety remains difficult to optimize because toxicity is closely associated with robust T-cell activation. Therefore, future studies should focus on developing programmable CAR-T and TCR-T systems capable of dynamically regulating activation intensity according to the tumor microenvironment rather than relying solely on constitutive receptor designs.

**FIGURE 5:**
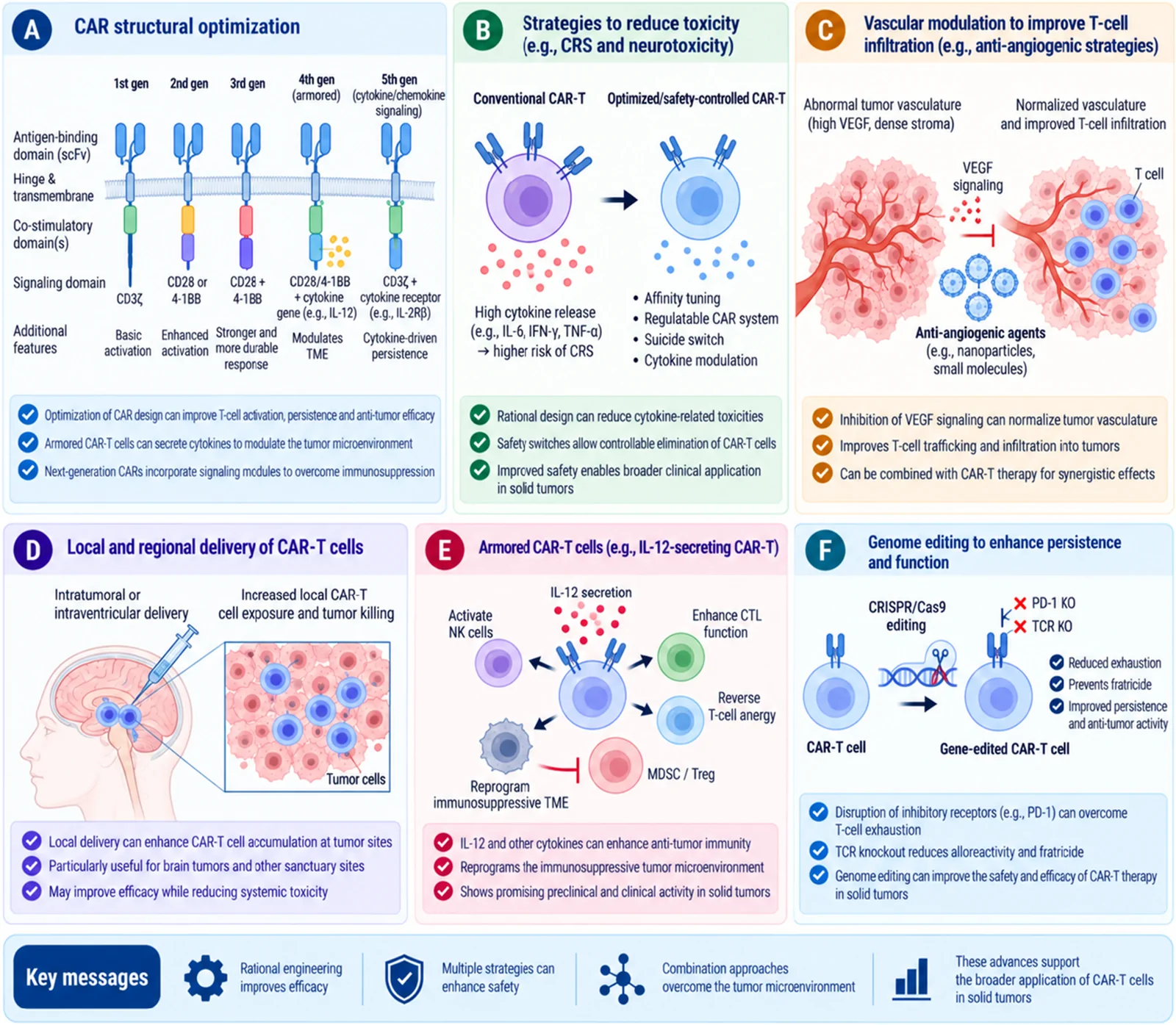
Engineering strategies and combination approaches to enhance the efficacy and safety of CAR-T-cell therapy in solid tumors. **(A)** Schematic overview of CAR structural optimization across successive receptor generations, including modifications of co-stimulatory and signaling domains. **(B)** Strategies to reduce treatment-related toxicity, including affinity tuning, controllable CAR systems, safety switches, and cytokine modulation. **(C)** Conceptual illustration of vascular modulation and anti-angiogenic approaches that may improve CAR-T-cell trafficking and infiltration into solid tumors. **(D)** Schematic representation of local and regional CAR-T-cell delivery to increase cellular exposure at tumor sites. **(E)** Cytokine-armored CAR-T cells, exemplified by IL-12-secreting CAR-T cells, which may enhance effector-cell activity and reshape the immunosuppressive tumor microenvironment. **(F)** Genome-editing strategies, including disruption of inhibitory receptors and endogenous TCR components, to improve CAR-T-cell persistence and function. This figure is an original schematic illustration independently created by the authors to summarize information from the cited literature. No previously published figure or graphical element was directly reproduced.

### 4.2 Tumor immune escape and resistance

Tumor immune escape means that tumor cells evade the recognition and attack of the immune system through various mechanisms, such as tumor antigen loss, downregulation of MHC-I molecules, and upregulation of immunosuppressive molecular ligands, so that they can survive *in vivo* and prolifize uncontrolled. A common stratery of tumor cells evading killing by engineered T cells is downregulated surface antigens or peptide-HLA complexes (150). In addition to antigen loss, disruption of the antigen delivery process can lead to immune escape. Tumors are prone to various mutations, during which not only the expression of antigens is altered, but also the stability of HLA heterozygosity and MHC molecules are affected. These changes further affect the processing and delivery of antigens, finally inhibiting the recognition and killing of tumor cells by T cells (151). Targeting multiple antigens is an effective strategy to avoid tumor antigen escape. Relevant clinical results have proved that bispecific CAR can reduce tumor recurrence caused by single antigen escape (152, 153). Many patients experienced relapse and progression after CD19 CAR-T therapy, and most of them experienced loss or downregulation of CD19. To prevent recurrence due to CD19 loss and to treat diseases with low CD19 expression, a clinical trial (NCT03233854) explored the therapeutic effect of CD19 and/or CD22 bispecific CARs and found that CD19 antigen loss is indeed a major cause of CAR-T resistance (154). Similarly, BCMA antigen loss was found in clinical treatments targeting BCMA (155). The results of a phase I clinical trial showed that BCMA-CD19 dual-targeted CAR-T cell therapy had favorable safety, pharmacokinetics, and antimyeloma activity in the early stages of treatment of high-risk patients with multiple myeloma (156). Several clinical trials are currently investigating the therapeutic efficacy and safety of BCMA/CD19 dual-targeted CAR-T cells. Current strategies mainly focus on targeting multiple tumor-associated antigens to prevent antigen escape. However, antigen evolution is a dynamic biological process driven by immune selection pressure rather than a single genetic event. Consequently, simply increasing the number of targeted antigens may delay but cannot completely prevent immune escape. Future approaches integrating real-time antigen monitoring, personalized neoantigen identification, and adaptive CAR or TCR redesign may provide more durable clinical responses.

### 4.3 Tumor heterogeneity

Tumor heterogeneity represents one of the major biological barriers limiting the efficacy and durability of adoptive T-cell therapy. Solid tumors exhibit heterogeneity at multiple levels, including interpatient variation, differences among metastatic lesions within the same patient, and diversity among individual tumor cells within a single lesion (157). These differences involve antigen expression profiles, genetic alterations, spatial distribution, and interactions with the tumor microenvironment, which collectively influence immune recognition and therapeutic response. Among these factors, antigen heterogeneity is particularly important for engineered T-cell therapies. Unlike hematological malignancies, where lineage-associated antigens such as CD19 and BCMA are relatively consistently expressed, solid tumors frequently contain antigen-positive and antigen-negative subpopulations. Consequently, CAR-T or TCR-T cells may eliminate antigen-expressing tumor cells while allowing antigen-low or antigen-negative clones to survive, resulting in disease recurrence and therapeutic resistance. This phenomenon is closely associated with immune escape mechanisms discussed above, particularly antigen loss and selective pressure induced by targeted therapy (150–155). In addition to antigen diversity, spatial heterogeneity within tumors creates distinct regional environments characterized by differences in oxygen availability, nutrient distribution, stromal composition, and immune cell infiltration. These variations can affect T-cell trafficking, activation, persistence, and cytotoxic function, thereby reducing the effectiveness of adoptive cell therapies in solid tumors. The interaction between tumor heterogeneity and the immunosuppressive tumor microenvironment further complicates therapeutic optimization. In addition to antigen diversity, spatial heterogeneity within tumors creates distinct regional environments characterized by differences in oxygen availability, nutrient distribution, stromal composition, and immune cell infiltration. These variations can affect T-cell trafficking, activation, persistence, and cytotoxic function, thereby reducing the effectiveness of adoptive cell therapies in solid tumors. The interaction between tumor heterogeneity and the immunosuppressive tumor microenvironment further complicates therapeutic optimization. Several strategies have been explored to overcome tumor heterogeneity, including multi-antigen targeting, dual CAR or tandem CAR designs, and personalized TCR-T approaches based on tumor-specific antigen identification. Multi-target recognition may reduce the probability of antigen-negative relapse, whereas individualized antigen discovery provides opportunities to develop patient-specific cellular products (78, 79, 117–119, 152, 153). However, increasing receptor complexity may also introduce challenges related to manufacturing, safety, and regulatory evaluation. Therefore, future development of adoptive T-cell therapy will require a balance between broader tumor recognition and precise control of specificity.

### 4.4 Tumor accessibility and tumor infiltration

Unlike hematological malignancies, the therapeutic efficacy of CAR-T and TCR-T in solid tumors is severely affected by physical barriers such as the tumor stroma, which limit the movement and penetration of engineered T cells into the tumor. The tumor vasculature system and extracellular matrix are key factors preventing T cell transport.

The uncontrolled proliferation and distant metastasis of tumors require a large amount of nutrients and oxygen. Tumor vasculature has different Pathological and physiological characteristics from the normal vascular system. The development of abnormal vasculature is closely related to the progression of malignant tumors, which also provides a new thinking about cancer treatment: targeting molecules related to tumor angiogenesis, destroying already generated blood vessels, and increasing the vascular permeability of tumor vessels, thus blocking the nutrient and oxygen supply or facilitate the penetration of other drugs into the tumor. Over the past decades, a number of anti-angiogenic drugs have been clinically approved, including the monoclonal antibodies: bevacizumab, ramucirumab; the receptor tyrosine kinase inhibitors: sorafenib, anlotinib, apatinib sunitinib, pazopanib and the endothelial cell proliferation inhibitors: thalidomide, angiostatin (157). With the development of nanotechnology, nanomedicine has also been used for targeted therapy of tumor vascular system. Some nanomaterials have pharmacological effects themselves. For example, gold nanoparticles can inhibit HUVEC proliferation by interfering with VEGF165 signaling (158), while silver nanoparticles can suppress VEGF-induced endothelial-cell proliferation through disruption of the PI3K/Akt pathway (159). These anti-angiogenic approaches conceptually illustrate how modulation of abnormal tumor vasculature may improve immune-cell access to solid tumors, as summarized in Figure 5C.

The extracellular matrix (EMC) is part of the matrix surrounding the tumor and consists of fibronectin, glycoproteins, polysaccharides, and proteoglycans. The increased expression and density of EMC components in malignant tumors, in particular the overproduction and deposition of hyaluronic acid and collagen, impede the penetration of therapeutic agents into the tumor (160). The expression profiles of EMC-related genes can be used as prognostic markers in many cancers. In pediatric osteosarcoma, the EMC remodeling genes desmoplakin, SPARCL1, biglycan, and PECAM are statistically different and can be used as prognostic factors in diagnosis (161). In gastric cancer, *ASPN, COL1A1, FN1, VCAN* and *MUC5AC* have the potential to serve as novel biomarkers for gastric cancer (162). Immunostaining and real-time imaging of human lung tumors revealed that activated T cells were predominantly active in loose fibronectin and collagen but poorly migrated in dense matrix regions (163). The limiting effect of the tumor extracellular matrix on therapy can be overcome by decreasing EMC stiffness, local administration, using chemokine receptors, or mobilizating other immune cells. Depletion of the tumor EMC can target hyaluronic acid (HA) by blocking its production or hyaluronidase digestion (164). 4-methylumbelliferone (4-MU) prevents HA formation in a variety of tumors by depleting the substrate required for hyaluronan synthesis, whereas hyaluronidase can directly degrade large HA polymers or simple polysaccharides (165, 166). The local administration strategy shortens the journey of engineered T cells to the disease site and minimizes interactions with normal tissue. In preclinical models of HER2-positive breast cancer brain metastasis, local and regional delivery of HER2-CAR T cells enhanced antitumor activity compared with systemic delivery (167). The principle that locoregional administration can increase CAR-T-cell exposure at anatomically restricted tumor sites is schematically illustrated in Figure 5D. *In situ* models of human pleural malignancies, intrapleural administration also demonstrated superior efficacy over intravenous administration (168). Based on these preclinical results, several clinical studies were conducted to evaluate the efficacy and safety of local delivery strategies (NCT02208362, NCT03389230, NCT03696030, NCT02414269). The use of chemokine receptors as guideposts for the transport of engineered T cells to the tumor site seems to improve the problem of insufficient T-cell infiltration. CXCR1 and CXCR2 are receptors for interleukin 8 (CXCL8), CXCR1 and CXCR2-modified CAR-T cells induced enhanced antitumor effects through IL8-directed migration into the tumor (169). Malignant pleural mesothelioma (MPM) secretes a large amount of chemokine CCL2. CAR-T cells expressing its receptor CCR2b compensated for the insufficient tumor-targeting localization of conventional CARs by binding to CCL2, showing a multifold increase in T cell tumor infiltration (170). The use of CAR-loaded cytokines also serves as a mobilizing agent for other immune cells, such as CAR-T cells expressing IL7 and CCL19 exhibit greater anti-tumor capacity than conventional CARs (171). Current strategies to improve T-cell infiltration primarily focus on overcoming individual physical or chemokine barriers. Nevertheless, the tumor stroma, abnormal vasculature, extracellular matrix remodeling, and immune suppression interact synergistically to restrict immune-cell trafficking. Therefore, future therapeutic strategies should combine vascular normalization, extracellular matrix remodeling, chemokine engineering, and local delivery to achieve efficient and sustained infiltration of engineered T cells into solid tumors.

### 4.5 Immunosuppressive microenvironment

The microenvironment of tumor cells is composed of invasive and resident cells, secretory factors and extracellular matrix. Infiltrating cells in the tumor microenvironment (TME) include a variety of cell types that can drive immunosuppression such as regulatory T cells (Treg), tumor-associated macrophages, myeloid-derived suppressor cells (MDSCs) (172). These immunosuppressive cells in turn drive tumor promoting factor, chemokines, growth factors. T cells in the tumor microenvironment also express many immune checkpoint factors such as PD-L1, CTLA-4, LAG3, the interaction of these components provides a suitable environment for the malignant proliferation of tumor cells (173). MDSC and Treg in the microenvironment can secrete TGF-β and interleukin 10 (IL-10) to inhibit the proliferation and activation of T cells and B cells (174). Therefore, inhibition of TGFβ, IL-10, and related cytokine transduction might be able to reverse the immunosuppression. It has been found that in triple-negative breast cancer, TGF-β can significantly impair the cytolytic activity, cytokine production, and cell proliferation of ROR1-CAR-T cells thereby reducing the viability of CAR T cells, while the TGF-β receptor kinase inhibitor SD208 protects CAR-T cells from the effects of TGF-β (175). Immunosuppressive cells can also be directly targeted to reverse the microenvironment. Drugs such as all trans retinoic acid (ATRA) and sunitinib that can inhibit MDSC are being explored for their potential as immunosuppressive cell inhibitors (176). In addition, the fourth generation of CAR-T cells: universal cytokine-mediated killing (TRUCK) T cells are being developed to enhance CAR-T cell activation against the immunosuppressive environment. A phase I study showed that IL-12-secreting CAR-T cells could enhance cytotoxic activity, persistence, and modulation of the tumor microenvironment (177). This strategy is conceptually summarized in Figure 5E, which illustrates how cytokine-armored CAR-T cells may enhance effector-cell activity and reshape the immunosuppressive tumor microenvironment (178). Although cytokine-armored CAR-T cells and checkpoint blockade have demonstrated encouraging preclinical activity, the tumor microenvironment remains highly heterogeneous among different tumor types and even between patients. Consequently, a universal strategy for overcoming immune suppression is unlikely to exist. Rational combination therapies integrating cellular engineering, immune checkpoint modulation, metabolic intervention, and stromal remodeling are expected to produce more consistent clinical benefits.

### 4.6 T-cell depletion

In chronic viral infections or tumors, T cells are converted to depleted T cells due to persistent antigenic stimulation. Depleted T cells exhibit dysfunction and diminished responses as evidenced by their diminished anti-tumor capacity, reduced cytotoxicity and effector cytokine release, impaired proliferative potential, and persistent expression of checkpoint molecules. T cell dysfunction and depletion is a major impediment to engineered T cell therapy (179). Clinically, lymphodepletion therapy is usually performed prior to infusing back T cells to increase T cell persistence (180). The results of several clinical studies have demonstrated that immune checkpoint inhibitors in combination with genetically engineered T cell therapy can effectively block the suppression of engineered T cells by immunosuppressive molecules. In a phase I study, by regional delivery of autologous mesothelin-targeted CAR-T in combination with pembrolizumab (anti-PD-1), patients achieved a median overall survival of 23.9 months, eight patients had stable disease for more than 6 months, and two patients demonstrated a complete metabolic response on PET scanning (181). In addition to combining with antibody drugs, T cells can be edited to become CAR-T cells targeting delivery of PD-1 scFv to block PD-1, which acts by paracrine or autocrine means, which improves safety and avoids toxicity associated with immune checkpoint inhibition (182).

CRISPR/Cas9 technology is an efficient and highly accurate gene editing tool that has become very popular in recent years. Because of its simplicity, flexibility, and effectiveness, CRISPR/Cas9 technology-mediated gene disruption and gene expression modulation have been applied to ameliorate the dysfunction of T cells. CRISPR/Cas9 technology has been used to knock down the gene encoding PD-1 on T cells to generate PD-1-deficient and T cell receptor-deficient mesothelin-specific CAR-T cells (183) (Figure 5J), blocking immune checkpoints at the gene editing level significantly reduced T-cell depletion. In addition to immune checkpoint molecules, CRISPR/Cas9 technology can also be used to modulate molecules that play a negative regulatory role in T-cell activation and proliferation such as SHP-1, an inhibitory protein tyrosine phosphatase. By co-transfecting primary T cells with Cas9: sgRNA and CAR expression plasmids, SHP-1 knockdown significantly improves CD133 CAR-T cells’ anti-tumor efficacy (184). CRISPR/Cas9 technology was used to disrupt the T-cell receptor alpha chain and remove CD52 from CAR19 T cells in a phase I clinical trial, and the results of this clinical trial demonstrated the feasibility, safety and therapeutic potential of CRISPR-engineered immunotherapy (185). Through CRISPR/Cas9 technology, IL12 was specifically expressed at the PDCD1 site of T cells, and T cell activation dependent expression of IL12 was realized, which enabled TCR-T cells to show more potent *in vivo* expansion capacity (186). In TCR-T technology, endogenous TCRs in T cells can also be knocked down by the CRISPR/Cas9 gene editing system, thus avoiding impaired TCR-T cell function caused by TCR chain mismatch (187). The molecular mechanisms that influence CAR-T cell depletion and differentiation are still poorly understood, fortunately, the development of CRISPR/Cas9 technology has also facilitated the screening of new targets and regulators of T cell depletion and memory cell formation. A genome-wide CRISPR knockdown screen identified a new gene that regulates T-cell incompetence: the PASA2. PASA2 knockdown CAR-T cells exhibited enhanced activation, cytokine production, and metabolic activity (188). Constructed an *in vitro* model of CD8 T-cell depletion by chronic stimulation, combined with a CRISPR/Cas9 genome-wide screen to generalize the epigenetic characteristics of T-cell depletion and advance the study of T-cell depletion (189). Katherine A. et al. induced CAR-T cell depletion by continuous antigen exposure and performed a CRISPR knockdown screen on CAR-T cells to identify two major regulators of T-cell effector function: MED12 and CCNC. They verified enhanced anti-tumor capacity of MED12-deficient and CCNC-deficient CAR-T cells (190). The application of cutting-edge technologies provides new hope and innovative strategies to improve T-cell overlay therapies. T-cell exhaustion is increasingly recognized as a complex developmental program involving transcriptional, metabolic, and epigenetic remodeling rather than simple checkpoint overexpression. Although CRISPR-mediated disruption of inhibitory genes has shown promising results, multiplex genome editing and epigenetic engineering may ultimately provide more durable restoration of T-cell fitness. Importantly, balancing enhanced persistence with long-term safety remains a major challenge for clinical translation.

### 4.7 The problems in manufacture

It is important to mention that while T-cell therapy is effective, the time required for its preparation and its high cost further limit its use in patients. Autologous T-cell therapy requires isolating each patient’s white blood cells and customizing specific T cells. The customization process is not 100% successful, and the cost of time puts patients with highly malignant tumors at greater risk (191). During tumor development, the patient’s own T-cells are likely to have been “domesticated” into incapacitated T-cells, which are not considered to be the best choice for T-cell therapy. The concept of ‘off-the-shelf’ allogeneic T cells has made it possible to offer T cell adoptive therapy as an off-the-shelf drug. Functioning T cells from healthy donors can provide a constant supply of high quality T cells for “T cell drugs”, no longer limited by the quality of the “raw material” (192). Allogeneic T cells are also expected to reduce costs through industrialization and scale-up. However, the allogeneic method also has its shortcomings. Most commonly, allogeneic T cells can cause life-threatening graft-versus-host disease (GVHD), and secondly, the persistence of these allogeneic T cells is further limited by the host immune system. These issues can be addressed by a series of genetic engineering edits to T cells, such as knockdown of the αβ T cell receptor (193). Despite the remarkable clinical success of adoptive T-cell therapy, several challenges continue to limit its broader application. Treatment-related toxicities, including cytokine release syndrome and neurotoxicity, remain major concerns, particularly for highly activated engineered T-cell products. In addition, limited T-cell persistence, functional exhaustion, immunosuppressive microenvironments, and therapeutic resistance represent important barriers that affect long-term clinical responses. To overcome these limitations, multiple strategies have been developed to improve the safety, specificity, and durability of adoptive T-cell therapies. These approaches include the identification of more appropriate tumor-specific neoantigens, multi-antigen targeting strategies to reduce immune escape, CRISPR/Cas9-based genetic modification to optimize T-cell function, and combination therapies designed to enhance antitumor activity. As summarized in Figure 6, successful future development of adoptive T-cell therapy will likely require the integration of improved antigen selection, cellular engineering, and rational therapeutic combinations.

**FIGURE 6:**
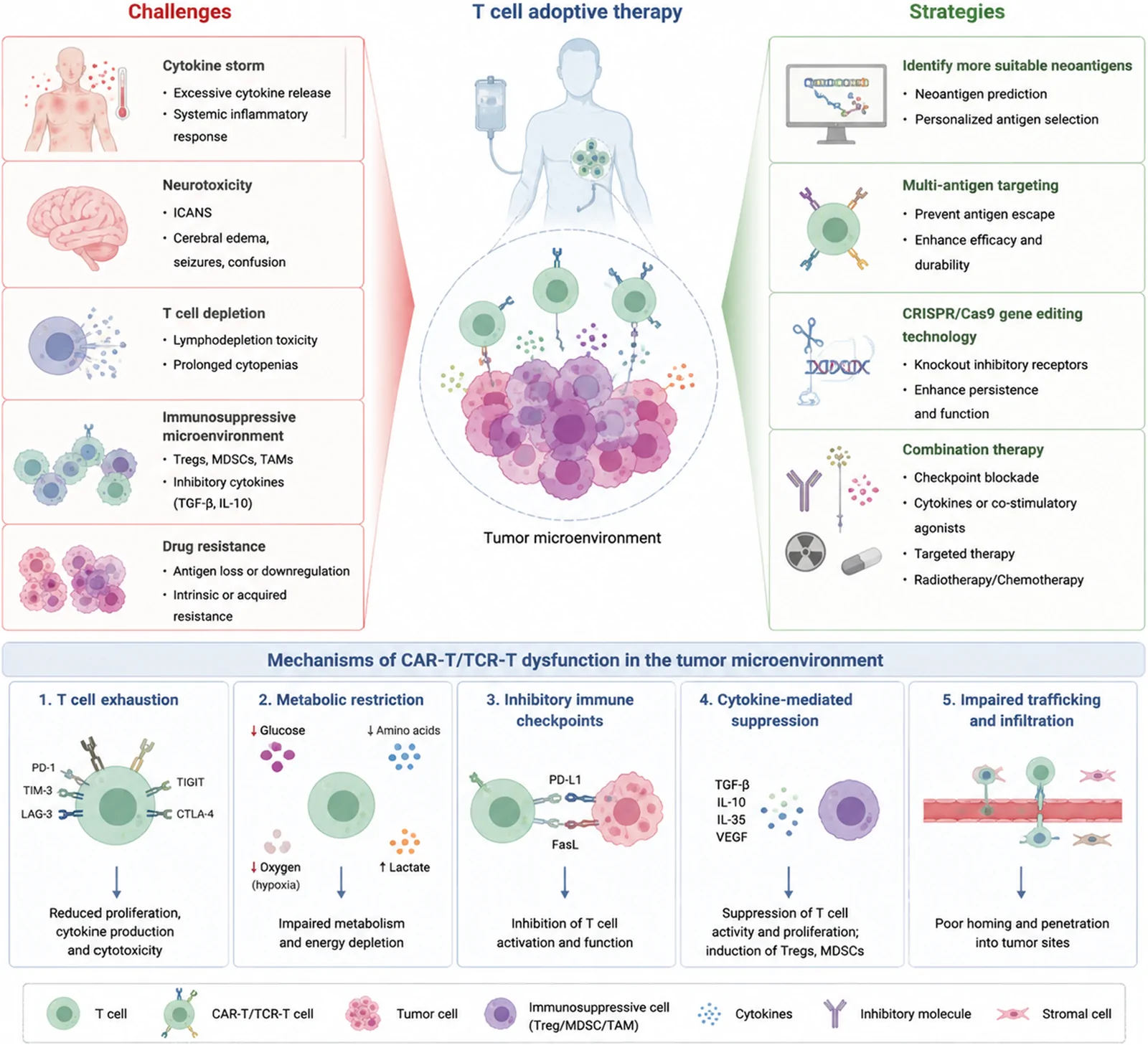
Major challenges and emerging strategies for improving T-cell adoptive therapy. This schematic summarizes the major limitations currently restricting the broader clinical application of T-cell adoptive therapy and the corresponding strategies developed to overcome these barriers. The left panel illustrates key challenges, including cytokine release syndrome, neurotoxicity, T-cell exhaustion or dysfunction, immunosuppressive tumor microenvironments, and therapeutic resistance. The right panel summarizes representative strategies aimed at improving therapeutic efficacy, including identification of more suitable neoantigens, multi-antigen targeting approaches, CRISPR/Cas9-mediated T-cell engineering, and combination therapies. This figure provides an integrated overview of how advances in antigen discovery, genetic engineering, and treatment optimization may address current limitations of adoptive T-cell therapy. This figure is an original schematic illustration independently created by the authors to summarize information from the cited literature. No previously published figure or graphical element was directly reproduced.

## 5 Emerging directions beyond conventional T-cell ACT

### 5.1 Expansion of ACT to alternative immune effector cells

Although CAR-T and TCR-T therapies currently dominate the field of adoptive cell therapy (ACT), increasing attention has shifted toward engineering alternative immune effector cells with complementary biological properties. Among these, chimeric antigen receptor-natural killer (CAR-NK) cells,CAR-macrophages (CAR-Ms), and CAR-regulatory T cells (CAR-Tregs) have emerged as promising next-generation ACT platforms.

CAR-NK cells possess several intrinsic advantages over conventional CAR-T cells. Unlike T lymphocytes, NK cells recognize malignant cells through both CAR-dependent and CAR-independent mechanisms and exhibit minimal risk of graft-versus-host disease (GvHD), making allogeneic “off-the-shelf” manufacturing feasible. Moreover, CAR-NK therapy is associated with lower incidences of cytokine release syndrome (CRS) and immune effector cell-associated neurotoxicity syndrome (ICANS), while maintaining encouraging antitumor activity in hematological malignancies. Recent advances in induced pluripotent stem cell (iPSC)-derived and cord blood-derived CAR-NK products further support scalable clinical production.

CAR-macrophages (CAR-Ms) represent another promising strategy, particularly for solid tumors. Unlike T cells, macrophages naturally infiltrate dense stromal tissues and hypoxic tumor regions that frequently limit CAR-T cell trafficking. CAR-Ms retain strong phagocytic activity and antigen-presenting capability while simultaneously remodeling the immunosuppressive tumor microenvironment by recruiting endogenous immune cells and promoting pro-inflammatory responses. These characteristics make CAR-M therapy particularly attractive for tumors characterized by abundant extracellular matrix deposition and poor T-cell infiltration.

Conversely, CAR-Tregs have emerged as an innovative platform for immune tolerance induction. Instead of eliminating target cells, CAR-Tregs suppress excessive immune activation in an antigen-specific manner through cytokine secretion, metabolic competition, and inhibitory receptor signaling. Their application has shown considerable promise in autoimmune diseases, transplantation tolerance, and graft-versus-host disease, offering a highly targeted alternative to systemic immunosuppression.

Collectively, these emerging immune cell platforms substantially broaden the scope of ACT beyond conventional T-cell engineering and are expected to complement existing CAR-T and TCR-T therapies according to disease-specific immunological requirements.

### 5.2 Diversification of gene delivery systems

Current clinical manufacturing of engineered immune cells relies predominantly on γ-retroviral and lentiviral vectors because of their high transduction efficiency and stable transgene expression. Alternatively, mRNA electroporation provides transient CAR expression and improved safety but often requires repeated administration due to limited persistence. Despite these advances, viral vector production remains expensive, technically demanding, and associated with concerns regarding insertional mutagenesis.

Consequently, non-viral gene transfer technologies have attracted increasing attention. Among these, the Sleeping Beauty (SB) and PiggyBac (PB) transposon systems represent the most extensively investigated alternatives. Both systems mediate stable genomic integration through transposase-dependent “cut-and-paste” mechanisms while avoiding viral vector production, thereby substantially reducing manufacturing costs and simplifying large-scale cell production. The Sleeping Beauty system has demonstrated favorable genomic integration profiles and has already entered early-phase clinical studies of CAR-T therapy. PiggyBac offers considerably larger cargo capacity and higher transposition efficiency, facilitating multiplex gene engineering, including simultaneous CAR expression, cytokine modulation, and immune checkpoint editing.

When combined with CRISPR/Cas9-mediated genome editing, these non-viral platforms provide a highly flexible strategy for generating next-generation engineered immune cells with improved safety, lower production costs, and enhanced therapeutic functions. As manufacturing technologies continue to mature, transposon-based engineering is expected to become an important complement—or even an alternative—to conventional viral vector approaches.

### 5.3 Expanding clinical applications beyond cancer and autoimmune diseases

Although ACT has achieved its greatest clinical success in oncology and autoimmune disorders, its therapeutic potential is rapidly expanding into other immune-related diseases.

Chronic viral infections constitute one of the most promising new indications. In human immunodeficiency virus (HIV) infection, CAR-T and TCR-engineered T cells targeting HIV envelope proteins or conserved viral epitopes have demonstrated prolonged antiviral activity and the ability to eliminate latent viral reservoirs in preclinical studies. Similarly, HBV-specific TCR-T and CAR-T therapies are being investigated for chronic hepatitis B virus (HBV) infection, aiming to eradicate infected hepatocytes while restoring antiviral immunity and reducing the risk of hepatocellular carcinoma.

Emerging evidence also supports the application of engineered immune cells in fibrotic diseases. Activated fibroblasts expressing fibroblast activation protein (FAP) have become attractive therapeutic targets. Experimental studies have shown that CAR-T cells targeting FAP can selectively eliminate pathogenic fibroblasts, reduce extracellular matrix deposition, and alleviate tissue fibrosis in cardiac, pulmonary, hepatic, and renal fibrosis models. Furthermore, CAR-M therapy may simultaneously enhance matrix degradation and promote tissue remodeling, suggesting a dual mechanism for reversing fibrotic progression.

These expanding applications illustrate that ACT is evolving from a cancer-focused immunotherapy into a versatile platform for treating chronic infectious diseases, fibrosis, and potentially other immune-mediated disorders.

## 6 Conclusion

Many clinical trials of ACT have been conducted since CAR-T cells demonstrated successful inhibition of hematological malignancy progression in 2008 (194). The clinical success of ACT in hematological malignancies, especially B-cell malignancies, has highlighted the therapeutic potential of engineered immune cells and attracted increasing attention toward their application in autoimmune diseases (195). However, despite remarkable advances, several challenges continue to limit the broader application of ACT. In hematological malignancies, antigen escape, limited T-cell persistence, immune-related toxicities, and disease relapse remain important obstacles. In solid tumors, additional barriers, including tumor heterogeneity, insufficient T-cell trafficking and infiltration, immunosuppressive tumor microenvironments, and lack of tumor-specific antigens, substantially restrict therapeutic efficacy.

TCR-T cell therapy has been widely explored in solid tumors because it can recognize intracellular tumor-associated antigens presented by major histocompatibility complex molecules and is therefore not restricted to surface antigen expression alone. However, issues including antigen specificity, tumor immune escape, and potential off-target effects remain critical considerations for further development. Therefore, future ACT strategies require not only improved antigen identification but also more precise engineering approaches to enhance safety, specificity, and long-term persistence.

Recent studies of ACT have focused on developing strategies to reduce treatment-related toxicity and improve T-cell function and persistence. For example, oncolytic viruses and radiation therapy can enhance T-cell recruitment and tumor homing, while combination therapies involving checkpoint inhibitors, cytokines, and other immunomodulatory approaches may improve T-cell activation and durability (17).

In addition, nanoparticle-based mRNA vaccines can promote the expression of target antigens and enhance CAR-T cell activity, providing a potential strategy to improve tumor targeting and therapeutic efficacy (196). Advances in gene-editing technologies, optimized CAR designs, multi-antigen targeting strategies, and combination approaches may further overcome current limitations.

Although ACT has transformed the treatment landscape of hematological malignancies and provided new opportunities for autoimmune diseases, several unresolved questions remain. Future studies should determine how to achieve optimal balance between T-cell activation and safety, how to maintain long-term persistence without inducing exhaustion, and how to overcome antigen heterogeneity and immunosuppressive environments in solid tumors. In addition, improving manufacturing consistency, reducing treatment costs, and developing more accessible therapeutic platforms will be essential for broader clinical application.

Looking forward, the next-generation of ACT is expected to evolve beyond conventional autologous CAR-T and TCR-T therapies. Emerging immune cell platforms, including CAR-NK cells, CAR-macrophages, and CAR-Tregs, are expanding the therapeutic repertoire by providing complementary mechanisms of action and improved safety profiles. Meanwhile, non-viral gene engineering technologies such as Sleeping Beauty and PiggyBac transposon systems offer more economical and scalable manufacturing strategies. Beyond oncology and autoimmune diseases, increasing evidence supports the application of ACT in chronic infectious diseases and fibrotic disorders. Together, these advances suggest that ACT is transitioning toward a broader, more versatile, and increasingly personalized immunotherapeutic platform.

Overall, optimizing T-cell engineering to achieve potent antitumor or immunomodulatory activity while minimizing toxicity represents a central goal for the next-generation of ACT. Continued integration of cellular engineering, gene-editing technologies, antigen discovery, and rational combination therapies will be critical for expanding the therapeutic potential of ACT beyond current clinical indications.

## Data Availability

The original contributions presented in the study are included in the article/Supplementary Material, further inquiries can be directed to the corresponding author.
