# Supplementary material for: Adoptive T Cell therapy for cancer and autoimmune disease: current advances, challenges, and future directions
Source: Oncol Rev. 2026 Sep 16;20:1905358. doi: 10.3389/or.2026.1905358 (PMC13625181; doi:10.3389/or.2026.1905358)
Supplement: Supplementary file 1 [file Table1.DOCX]

Figures 1 Structural characteristics and mechanism of CAR-T and TCR-T. The CAR has undergone five generations of improvements. The first generation CARs had only intracellular CD3-ζ signaling molecules. In the second generation, CD28 or 4-1BB co-stimulatory region was added to integrate CD3-ζ molecules. The third generation adds two different co-stimulatory domains (CD28-4-1BB/ICOS-4-1BB) to the second generation. The fourth generation, called TRUCKs or armored CARs, is paired with constitutionally expressed chemokines. The fifth generation also added the intracellular domain of the cytokine receptor (IL-2Rβ) on the basis of the second generation. IL-2Rβ promotes cell proliferation and increases persistence by activating the JAK-STAT signaling pathway. T cell therapy mediates tumor killing in several ways: ① perforin and granase ②CAR T or TCR-T activate secreted cytokines ③Fas-FasL axis

Figure 2 Clinical outcomes and representative design features of CAR-T-cell therapies for hematological malignancies. (A–C) Best overall responses in all evaluable patients and in patients with follicular lymphoma or marginal zone lymphoma. Responses were assessed by an independent radiology review committee. (D) Kaplan–Meier estimate of the duration of remission, with censoring at subsequent allogeneic stem-cell transplantation. (E) Duration of remission without censoring at subsequent allogeneic stem-cell transplantation. (F) Relapse-free survival, with censoring at subsequent allogeneic stem-cell transplantation. (G) Overall survival. CR, complete remission; CRi, complete remission with incomplete hematologic recovery; NE, not estimable; NR, not reached. (H) Comparison of representative first-, second-, and third-generation anti-CD19 CAR architectures. (I) Structural organization of the lisocabtagene maraleucel CAR, including the CD19-binding domain, 4-1BB co-stimulatory domain, and CD3ζ signaling domain. (J) Schematic overview of the lisocabtagene maraleucel manufacturing process, including leukapheresis, separate selection and activation of CD4+ and CD8+ T cells, transduction, expansion, formulation, and administration.

Figure 3 Clinical outcome after T cell treatment. (A), CT scans showed a reduction of primary tumor lesion (red line) before (baseline) and after CAR T EGFR cell treatment in patient 13. (B), MRI scans showed a reduction of metastatic lesions in the liver 1 month after CAR T EGFR celltreatment in patient 1. Red arrows indicate metastatic lesions in the liver. (C), IHC of metastatic lesions in the liver in patient 1. (D), CART-meso expansion and persistence in the peripheral blood was detected by qPCR. (E), Peripheral blood expansion of CART-meso cells is shown for each patient and grouped by cohort. Peak CART-meso levels observed among patients in cohort 1 (991.76 copies/μg, patient 1-02) are depicted by a blue line in each plot for reference. (F), Diagram of the recombinant retroviral vector MSGV1AIB used to engineer human lymphocytes. (G), Computed tomography (CT) images of liver metastasis in patient 4 taken at pretreatment, 1 month, and 10 months after treatment with TCR-engineered T cells. (H), Size of liver and axillary tumors and tempo of regression of tumor sites in patient 4. Day 0, beginning of treatment. (I), CT images of hilar lymph node metastasis in patient 14; pretreatment, day 0, and 2 months and 12 months after treatment. The white arrow indicates the mass in the lung hilum. (J), Size of tumor and tempo of regression in patient 14. (K), Histopathological features of dermatitis from patient 6. Hematoxylin–eosin (HE) and immunohistochemical stainings of MART-1 and CD3 were carried out on skin biopsies from the back of affected and unaffected skin, taken 4 days after development of dermatitis (6 days after cell infusion). Loss of melanocytes and MART-1 expression and infiltration of CD3+ T cells along the dermoepidermal junction can be seen in the affected skin compared to the unaffected skin (magnification ×200).

Figure 4 The cutting-edge applications of T-cell adoptive therapy across different cancer types. CAR-T cell therapy was initially successful in hematologic tumors, and its therapeutic effect in a variety of solid tumors has been gradually explored. In melanoma, in addition to targeting antigens on tumor cells, attacking the vasculature with CAR-T can also inhibit melanoma. Brain tumors have a more complex immune environment than other tissues, and local administration strategies can promote the effectiveness of T cell therapy in brain tumors. In prostate cancer, the target antigen is mainly PSMA, which is a prostate specific membrane antigen. In breast cancer, probiotics are used to label tumors and direct CAR T cells to target them. DLL3 is an emerging target for small cell lung cancer, and T-cell adoptive therapies targeting DLL3 are under clinical investigation. Cervical cancer is a cancer explicitly caused by HPV virus infection, so CAR-T therapy has been used to target HPV cancer proteins E6 and E7. Both pancreatic cancer and gastric cancer belong to digestive system malignancies, and CLND18.2 is their common tumor antigen, which has shown good results in clinical trials. Since T cells and B cells are highly correlated with autoimmune diseases, the application of immune cell therapy in autoimmune diseases is gradually developing. CD19 and BCMA double-targeted CAR-T can not only induce B cell depletion, but also target long-life plasma cells, which has the potential to completely remove autoantibodies to achieve better clinical results. This figure was adapted and redrawn based on previously published studies cited in the corresponding section.

Figure 5 Emerging strategies to overcome limitations of adoptive T-cell therapy.(A), Schematic of the recombinant lentiviral vectors encoding the prototype anti-CD19 CAR (CD19-BBz(71); also termed CTL019) or variants generated in this study.(B), Predicted tertiary structures of the CD19-BBz CAR variants in the PyMOL molecular graphics program. αCD19, anti-CD19 scFv; EC, extracellular domain; IC, intracellular domain; TM, transmembrane domain. (C), Representative PET–CT scans of six patients before and after CAR T cell treatment showing durable complete remission. Red arrows indicate sites of lymphoma. (D), CT scans showing long-term remission in patient BZ004 treated with CD19-BBz(86) CAR T cells at a dose of 3 × 106 cells. Circles indicate sites of lymphoma. Scale bars, 10 cm. (E), TEM images obtained from purified fractions collected after sucrose density gradient of Ag-NPs synthesized using B. licheniformis (F), Ag-NPs or PEDF inhibit VEGF-induced migration of endothelial cells. (G), Illustration of a mouse brain orthotopically engrafted with BBM1-eGFP/ffluc cells and treated by intratumoral (intracranial, i.c.) delivery of CAR T cells. (H), Local intratumoral delivery of HER2-CAR T cells eradicates xenograft models of breast cancer brain metastasis. (I), Secretion of IL-12 by CAR-modified T cells may improve cytotoxicity of CAR + T cells and reverse anergy in tumor infiltrating lymphocytes. (J), Flow cytometric analysis of CAR, PD-1, and TCR surface expression in MPTK-CAR-T cell products.

Figure 6 Major challenges and emerging strategies for improving T-cell adoptive therapy.This schematic summarizes the major limitations currently restricting the broader clinical application of T-cell adoptive therapy and the corresponding strategies developed to overcome these barriers. The left panel illustrates key challenges, including cytokine release syndrome, neurotoxicity, T-cell exhaustion or dysfunction, immunosuppressive tumor microenvironments, and therapeutic resistance. The right panel summarizes representative strategies aimed at improving therapeutic efficacy, including identification of more suitable neoantigens, multi-antigen targeting approaches, CRISPR/Cas9-mediated T-cell engineering, and combination therapies.This figure provides an integrated overview of how advances in antigen discovery, genetic engineering, and treatment optimization may address current limitations of adoptive T-cell therapy. This figure was adapted and redrawn based on previously published studies cited in the corresponding section.
