# Supplementary material for: Adoptive T Cell therapy for cancer and autoimmune disease: current advances, challenges, and future directions
Source: Oncol Rev. 2026 Sep 16;20:1905358. doi: 10.3389/or.2026.1905358 (PMC13625181; doi:10.3389/or.2026.1905358)
Supplement: Supplementary file 2 [file Table2.DOCX]

| Factors | TCRs | CARs |
| --- | --- | --- |
| Sructure | Complex transmembrane receptors, ten ITAMs, needs Co-stimulation transmission. | Artificial single strand, three ITAMs. Contains Co-stimulation transmission. |
| MHC restriction | Yes | No |
| Typical affinity | K_d_ 1 -100μM | K_d_ 1 nM - 1μM |
| IS formation | Organized | Disorganized |
| FDA approval | Yes | No |
| Antigen types | Intracellular and extracellular | Only surface antigen |

Table 1 The structural differences between CAR and TCR

Table 2 Representative registered clinical trials of CAR-T therapy for hematological malignancies, grouped by target antigen.

| Target antigen | Disease/indication | Clinical phase | Trial identifier | Study period | Main objective(s) |
| --- | --- | --- | --- | --- | --- |
| CD19 | R/R HLM | I/II | NCT04790747 | 2021-2025 | Safety and efficacy, |
|  | CD19-expressing Malignancies | I | NCT04649112 | 2021-2023 | Safety |
|  | B-Cell Lymphoma and B-ALL | II | NCT05281809 | 2022-2037 | Feasibility |
|  | R/R Non-Hodgkin Lymphoma | I | NCT04545762 | 2020-2023 | Safety and feasibility |
|  | R/R MM | I | NCT04796688 | 2021-2024 | Safety and efficacy |
| BCMA | R/R M/M | I/II | NCT04271644 | 2019-2024 | Efficacy and safety |
|  | High-risk NDMM | II | NCT05846737 | 2023-2028 | Safety and efficacy |
|  | R/R MM involving CRISPR-Edited CAR-T cells | I | NCT05722418 | 2023-2027 | Safety and best dose |
|  | MM and AL amyloidosis | I | NCT04720313 | 2021-2026 | Dose escalation and safety |
|  | R/R Plasma Cell Blood Tumors | Early phase I | NCT06067581 | 2023-2025 | Safety and efficacy |
|  | High-Risk SMM | II | NCT05767359 | 2023-2040 | Safety and effectiveness |
| CD7 | Hematologic Malignancies | II | NCT05454241 | 2022-2025 | Efficacy, safety and PK |
|  | R/R CD7-positive T-ALL/lymphoma | I | NCT05212584 | 2022-2024 | Safety and tolerability |
|  | CD7-positive R/R T-ALL/LBL/AML | Early phase I | NCT05907603 | 2023-2025 | Safety and pharmacokinetics |
|  | R/R Lymphoid Hematologic Malignancies | I | NCT05170568 | 2021-2024 | Safety and tolerability |
| GPRC5D | R/R MM or PCL | II | NCT05219721 | 2022-2024 | Safety and efficacy |
| CD70 | CD70 Positive Malignant Hematologic | Early phase I | NCT04662294 | 2011-2027 | Safety |
| IM21 | R/R MM | Early phase I | NCT05478343 | 2021-2023 | Safety and efficacy |
| CD28 | Hematologic Malignancies | Early phase I | NCT05239689 | 2022-2024 | Safety and efficacy |
| CLL1 | CLL1-positive Hematological Malignancies | Early phase I | NCT05252572 | 2022-2024 | Safety and effectiveness |
| CS1 | R/R MM | I | NCT03710421 | 2019-2023 | Side effects and best dose |
| CD20 | R/R B Cell Non-Hodgkin Lymphomas | I/II | NCT03277729 | 2017-2037 | Best dose and safety |
| CD138 | R/R MM | I | NCT03672318 | 2029-2028 | Efficacy |
| CD123 | Hematologic and lymphatic malignancies | I | NCT04230265 | 2020-2025 | Safety, side effects, harmlessness, therapeutical benefit |
| ROR1 | Advanced Hematologic and Solid Tumor Malignancies | I | NCT05694364 | 2023-2026 | Dose escalation/Dose expansion study |
| NKG2D | R/R AML/MDS | I | NCT04167696 | 2019-2035 | Recommended dose |

Abbreviations: R/R, relapsed/refractory; B-ALL, B-cell acute lymphoblastic leukemia; MM, multiple myeloma; NDMM, newly diagnosed multiple myeloma; SMM, smoldering multiple myeloma; PCL, plasma cell leukemia; T-ALL, T-cell acute lymphoblastic leukemia; LBL, lymphoblastic lymphoma; AML, acute myeloid leukemia; MDS, myelodysplastic syndrome.

Table 3 Representative clinical trials of CAR-T targeting solid tumors

| Antigen | Tumor type | Phase | NCT number | Time | Main objective(s) |
| --- | --- | --- | --- | --- | --- |
| CEA | CEA-positive Advanced/Metastatic Malignant Solid Tumors | I | NCT06010862 | 2023-2026 | Safety and tolerability |
| CD70 | CD70-positive Advanced/Metastatic Solid Tumors | I | NCT06010875 | 2023-2026 | Safety and efficacy |
| EpCAM | Advanced Solid Tumors | I | NCT02915445 | 2026-2023 | Safety  and efficacy |
| MUC1-C | Advanced or Metastatic epithelial derived Solid Tumors | I | NCT05239143 | 2022-2039 | Safety, tolerability and response |
| ROR1 | ROR1+ R/R TNBC or NSCLC | I | NCT05274451 | 2022-2026 | Safety and tolerability |
| HER-2 | HER2 Positive Solid Tumors | I | NCT03740256 | 2020-2038 | Safety and efficacy |
| CLDN6 | CLDN6-positive relapsed or refractory advanced solid tumors | I/II | NCT04503278 | 2020-2037 | Safety and efficacy |
| B7-H3 | Pediatric Patients With R/R B7-H3+ solid tumors. | I | NCT04897321 | 2022-2027 | Safety and MTD |
| GD2 | High Risk and/or R/R Neuroblastoma or Other GD2-positive Solid Tumors | I/II | NCT03373097 | 2018-2027 | Safety and efficacy |
| CLDN18.2(CT041) | CLDN18.2 expression-positive, advanced gastric/esophagogastric conjugate adenocarcinoma | I/II | NCT04581473 | 2020-2038 | Efficacy, safety and pharmacokinetics |
| CLDN18.2 /Cytokines | Advanced Solid Tumors | I | NCT05393986 | 2022-2024 | Safety, tolerability, preliminary efficacy and pharmacokinetics |
| GPC3 | GPC3-Positive Solid Tumors | I/II | NCT05120271 | 2022-2039 | Safety and RP2D |
| MSLN(UCLMM802) | Mesothelin-positive Advanced Malignant Solid Tumors | I | NCT05848999 | 2023-2025 | Safety, tolerability and preliminary efficacy |
| NKG2D | R/R NKG2DL+ Solid Tumors | I | NCT05131763 | 2021-2023 | Safety and clinical activity |
| HLA-G(IVS3001) | Advanced HLA-G-Positive Solid Tumors | I/II | NCT05672459 | 2023-2029 | Safety, tolerability, pharmacokinetics and clinical activity |
| EGFR806 | Recurrent/Refractory Solid Tumors | I | NCT03618381 | 2019-2038 | Safety, feasibility, and efficacy |
| B7-H3(TAA06) | TAA06-positive advanced solid tumor | I | NCT05190185 | 2021-2023 | Safety, tolerability, distribution, proliferation and persistence |
| IL-15 Armored GPC3-specific CAR | Pediatric Solid Tumors | I | NCT04377932 | 2021-2040 | Maximum tolerated dose, side effect, peisistance |
| TGFβR-KO CAR-EGFR | Advanced EGFR-positive Solid Tumors | I | NCT04976218 | 2022-2024 | Anti-tumor activities and safety profiles |
| Anti-PD-L1/Anti-CD22 | Solid Tumors | I | NCT04556669 | 2020-2025 | Adverse reaction, pharmacokinetics, and the outcomes |
| VEGFR1 and PD-L1 | Pleural or Peritoneal Metastases | I | NCT05477927 | 2022-2024 | Safety and effectiveness |
| GD2/PSMA | GD2 and PSMA positive tumor | I/II | NCT05437315 | 2022-2026 | Feasibility, safety and efficacy, persistency |
| CD40 /MSLN | MSLN Positive Advanced/Metastatic Solid Tumors | I/II | NCT05693844 | 2023-2025 | Dose limiting toxicities, maximum tolerated dose |

Table 4 Table 4 Registered TCR-T clinical trials targeting solid tumors

| Antigen | Tumor type | HLA | Phase | NCT number | Time | Goals |
| --- | --- | --- | --- | --- | --- | --- |
| NY-ESO-1 | Advanced Solid Tumors | HLA-A2+ | I | NCT05881525 | 2023-2025 | safety and efficacy |
| NY-ESO-1 (TAEST16001) | Soft Tissue Sarcoma | HLA-A * 02:01 | I | NCT04318964 | 2020-2024 | safety, tolerance, PK, PD characteristics, and preliminary efficacy |
| NY-ESO-1/ dnTGFbetaRII | Advanced Malignancies | HLA-A2.1 | I/II | NCT02650986 | 2017-2032 | safety and feasibility, T cell differentiation that correlates with higher anti-tumor responses |
| MAGE-A4/8 | Solid tumors | HLA-A*02:01 | I | NCT03247309 | 2018-2024 | safety |
| MAGE-A1(TK8001) | Advanced-Stage/Metastatic MAGE-A1+ Solid Tumors | HLA-A*02:01 | I/II | NCT05430555 | 2022-2037 | Safety, tolerability and anti-tumoral activity |
| MAGE-A3/A6 | Adults With Advanced Cancers | HLA-DPB1*04:01 | I | NCT03139370 | 2017-2023 | safety and Efficacy |
| KK-LC-1 | KK-LC-1 Positive Epithelial Cancers | HLA-A01:01 | I | NCT05035407 | 2022-2026 | safety of different doses of KK-LC-1 TCR T cells plus aldesleukin |
| KK-LC-1 or neoantigens | advanced lung cancer or other solid tumors | MHC-A11 | I | NCT03778814 | 2018-2033 | safety, tolerance and preliminary efficacy |
| KRAS G12V | Advanced or Metastatic Solid Tumor | HLA-A*11:01 | I/II | NCT06105021 | 2023-2029 | safety , tolerability , OBD and RP2D |
| TP53 R175H Mutation | TP53 R175H mutant solid tumors | HLA-A*02:01 | I | NCT05877599 | 2023-2039 | Safety and Preliminary Anti-tumor Activity |
| KRAS/TP53/EGFR | relapsed/refractory solid tumors | HLA-A/HLA-C/HLA-DRB | I/II | NCT05194735 | 2022-2029 | DLT and MDT or RP2D |
| MAGE-A1, HPV16 E7, PRAME | Locally Advanced (Unresectable) or Metastatic Solid Tumors | HLA-A*02:01, HLA-C*07:02, | I | NCT05973487 | 2023-2026 | Safety, preliminary efficacy of single and repeat dose regimens of TCR'Ts as monotherapies and as T-Plex combinations |
| IMA203/IMA203CD8 | Recurrent and/or Refractory Solid Tumors | HLA-Dextramer | I | NCT03686124 | 2019-2028 | safety and tolerability |
| NY-ESO-1 and/or LAGE-1a | Advanced Solid Tumors | HLA-A*02 | I | NCT04526509 | 2020-2025 | Safety and RP2D |
| PRAME | metastatic/unresectable tumors | HLA-A2 | I/II | NCT04262466 | 2020-2026 | safety and efficacy |
| NY-ESO-1 and/or LAGE-1a | Solid Tumors | HLA-A*02 | II | NCT03967223 | 2019-2026 | safety and efficacy |

Table 5 Clinical trial of CAR-T cells for autoimmune diseaseable

| CAR-Antigen | Condition | Phase | NCT number | Time | Goals |
| --- | --- | --- | --- | --- | --- |
| CD19 | RSSc, R SS, RDM, Refractory the antineutrophil cytoplasmic antibody-associated vasculitides | I | NCT06056921 | 2023-2026 | efficacy and safety |
|  | R/M S SLE | I/II | NCT06106906 | 2023-2026 | Safety and tolerability |
|  | Refractory/Moderate-to-severe SLE | I/II | NCT06106893 | 2023-2026 | Safety and Efficacy((CAR-γδT Cells)) |
| CD19 | E/R AD | Unknow | NCT05859997 | 2023-2025 | Safety and Efficacy |
|  | Moderate to Severe Active SLE | I | NCT05765006  （Relma-cel） | 2023-2025 | safety tolerability pharmacokinetics and pharmacodynamics of Relma-cel |
|  | RLN | I | NCT05938725 | 2023-2026 | Incidence adverse events (AEs) and laboratory abnormalities; Frequency of dose limiting toxicities at each dose level |
|  | SLE or AIT | Early Phase 1 | NCT05930314  (CNCT19) | 2023-2025 | ate the safety and preliminary efficacy |
|  | S/R SLE | I/II | NCT05798117  (YTB323) | 2023-2026 | safety, efficacy and cellular kinetics |
|  | ASLE | I/II | NCT06121297  (CABA-201) | 2023-2027 | Safety and Efficacy |
| BCMA（Descartes-08） | SLE | II | NCT06038474 | 2023-2026 | safety and tolerability |
| BCMA/CD19 | R/R SLE | I | NCT05474885 | 2022-2025 | safety and tolerability |
| CD19-BCMA | RSLE | I | NCT05846347  （GC012F） | 2023-2025 | the recommended phase II dose of GC012F injectioni |
| BCMA(NXC-201) | ALA | 1b | NCT06097832 | 2024-2039 | safety and efficacy |
| BCMA | NMOSD、MG、CIDP 、IMNM | Early Phase 1 | NCT04561557 | 2020-2024 | Safety and Efficacy |
| CD7 | CD  UC  Dermatomyositis  Still Disease | Early Phase 1 | NCT05239702 | 2022-2024 | safety and effectiveness |
| CD19/BCMA | R POEMS, SAmyloidosis, AIHA, and Vasculitis | Early Phase 1 | NCT05263817 | 2021-2024 | Safety and Effectiveness |
| FKC288 | R/RS ALA | I | NCT05978661 | 2023-2026 | Evaluate the safety, explore the recommended phase II dose |
| CD19/BCMA | RSS | Early Phase 1 | NCT05085431 | 2023-2024 | Safety and Tolerability |
| CD19/BCMA | Refractory Scleroderma | Early Phase 1 | NCT05085444 | 2021-2024 | safety and effectiveness |
| CD19 / CD20 | R/R AQP4-IgG Seropositive NMOSD | I | NCT03605238 | 2018 | Safety and Efficacy |
